# Oxidative stress impairs the Nur77‐Sirt1 axis resulting in a decline in organism homeostasis during aging

**DOI:** 10.1111/acel.13812

**Published:** 2023-03-07

**Authors:** Yang Yu, Xiaoyu Song, Xiaoxun Wang, Lixia Zheng, Guojing Ma, Weiwei Liu, Han Su, Xiyan Liu, Tingting Liu, Liu Cao, Difei Wang

**Affiliations:** ^1^ Health Sciences Institute China Medical University Shenyang China; ^2^ Key Laboratory of Medical Cell Biology, College of Basic Medical Sciences China Medical University Shenyang China; ^3^ Department of Medical Oncology The First Hospital of China Medical University Shenyang China; ^4^ Department of Gerontology Shengjing Hospital of China Medical University Shenyang China

**Keywords:** aging, kidney injury, Nur77, oxidative stress, Sirt1

## Abstract

Sirt1 is an NAD^+^‐dependent deacetylase that protects against premature aging and cell senescence. Aging accompanied by oxidative stress leads to a decrease in Sirt1 levels and activity, but the regulatory mechanism that connects these events remains unclear. Here, we reported that Nur77, which shares similar biological pathways with Sirt1, was also decreased with age in multiple organs. Our in vivo and in vitro results revealed that Nur77 and Sirt1 decreased during aging and oxidative stress‐induced cell senescence. Deletion of *Nr4a1* shortened the lifespan and accelerated the aging process in multiple mouse tissues. Overexpression of *Nr4a1* protected the Sirt1 protein from proteasomal degradation through negative transcriptional regulation of the E3 ligase MDM2. Our results showed that Nur77 deficiency markedly aggravated aging‐related nephropathy and elucidated a key role for Nur77 in the stabilization of Sirt1 homeostasis during renal aging. We proposed a model wherein a reduction of Nur77 in response to oxidative stress promotes Sirt1 protein degradation through MDM2, which triggers cell senescence. This creates additional oxidative stress and provides positive feedback for premature aging by further decreasing Nur77 expression. Our findings reveal the mechanism by which oxidative stress reduces Sirt1 expression during aging and offers an attractive therapeutic strategy for targeting aging and homeostasis in organisms.

## Graphical abstract

Nur77 declines with aging and in response to ROS stimulation, which leads to the loss of Sirt1 via MDM2‐mediated proteasomal degradation. These events increase p53 stability and activation, which in turn further downregulate the expression of Nur77 and Sirt1 and accelerate the aging process in the kidney.

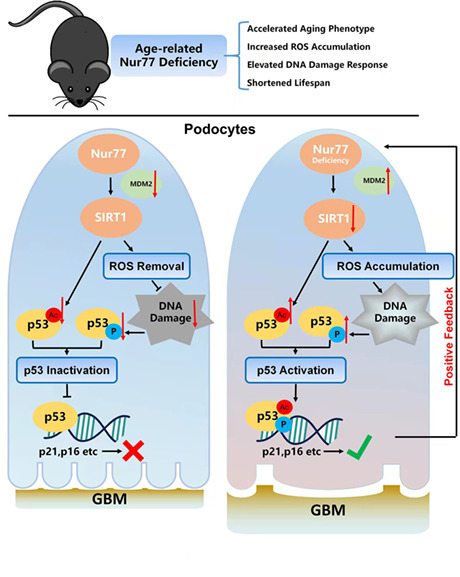

**Abbreviations**
ACR: ratio of ALB to urinary creatinine
ALB: urinary albumin
AUC: area under the curve
BUN: blood urea nitrogen
BW: body weight
CCr: creatinine clearance rate
CHX: cycloheximide
CQ: chloroquine
CVF: collagen volume fraction
DCFH‐DA: 2′,7′‐dichlorofluorescein diacetate
DHE: dihydroethidium
DMSO: dimethyl sulfoxide
FBG: fasting blood glucose
GSH‐PX: glutathione peroxidase
IB: immunoblot
IHC: immunohistochemistry
IP: immunoprecipitation
MA/G: mesangial area glomerulus
MG‐132: Z–Leu–Leu–Leu–al
MO: month‐old
NAC: *N*‐acetyl‐l‐cysteine
OS: serum extracted from aged mice
Res: resveratrol
ROS: reactive oxygen species
SCr: serum creatinine
SOD: superoxide dismutase
TAG: triglyceride
T‐CHO: total cholesterol
TUNEL: terminal deoxynucleotidyl transferase dUTP nick end label
Ub: ubiquitin
UV: urine volume
WT: wild‐type
YS: serum extracted from young mice

## 1 INTRODUCTION

Aging is a biological phenomenon in which the structure and function of organisms decline with increasing age (Lopez‐Otin et al., 2013). The Sir2 protein and its homologs belong to the sirtuin family of protein deacetylases and are collectively known to extend the lifespan in various species (Imai & Guarente, 2014). Of all the sirtuins, Sirt1 is the most extensively studied member. Sirt1 deacetylates key histone residues of multiple protein targets, including p53, forkhead box O 1 and 3 (FoxO1/3), peroxisome proliferator‐activated receptor gamma coactivator 1‐alpha (PGC‐1α), and nuclear factor kappa B (NF‐κB) (Bi et al., 2019; Gomes et al., 2013; Mouchiroud et al., 2013; Wellman et al., 2017). By affecting transcriptional activation, Sirt1 is involved in the regulation of a broad range of vital aging‐related biological pathways, including DNA repair and apoptosis, cell stress responses, and glucose and insulin homeostasis (Meng et al., 2020). Furthermore, Sirt1 declines with age, which disrupts the homeostasis of multiple organs and accelerates the aging process (Donato et al., 2011). However, the mechanism of the reduction in Sirt1 during aging in mammalian systems has remained unclear.

Another factor that shows an intrinsic relationship with aging is the overproduction of reactive oxygen species (ROS) (Szilard, 1959). During the aging process, multiple age‐related organ dysfunctions are associated with ROS accumulation. High levels of ROS hamper the repair of damaged nuclear and mitochondrial DNA at multiple steps and contribute to genomic instability (Li et al., 2021). There are mutual effects between oxidative stress and Sirt1 during the aging process. For example, moderate overexpression of Sirt1 protects against oxidative stress by inducing the major intracellular antioxidant catalase (Alcendor et al., 2007). Conversely, an increase in ROS can damage the protein expression and enzymatic activity of Sirt1 (Salminen et al., 2013). An increase in oxidative stress and a reduction in Sirt1 protein have been shown to occur in senescent cells, but the mechanisms that regulate the crosstalk between them during aging are still unclear.

The orphan nuclear hormone receptor Nur77 (also called TR3) has been implicated in similar biological pathways involved in aging. Nur77 belongs to the NR4A subgroup of nuclear hormone receptors and has emerged as an important regulator of the inflammatory response, metabolic homeostasis, and oxidative stress (Li et al., 2018). In macrophages, Nur77 helps to repress the transcription of proinflammatory factors, leading to a systemic decrease in the inflammatory response in elderly mice (Koenis et al., 2018). The overexpression of Nur77 in melanoma cells prevents ROS accumulation by binding to mitochondrial trifunctional protein β subunit (TPβ) to avoid NADPH depletion and maintain GSH levels (Li et al., 2018). Exactly how Nur77 is connected with other pathways and molecular mechanisms involved in aging is not understood.

Here, we investigated the internal regulatory mechanism that links increasing ROS levels and decreasing Sirt1 protein levels during aging. We found that Nur77 declined during aging and in response to ROS stimulation, which led to the loss of Sirt1 via MDM2‐mediated proteasomal degradation. These events increased p53 stability and activation, cell senescence, and ROS accumulation, which in turn further downregulated the expression of Nur77 and Sirt1 and accelerated the aging process.

## 2 RESULTS

### 2.1 Nur77 deficiency accelerates the aging process in multiple organs

**FIGURE 1:**
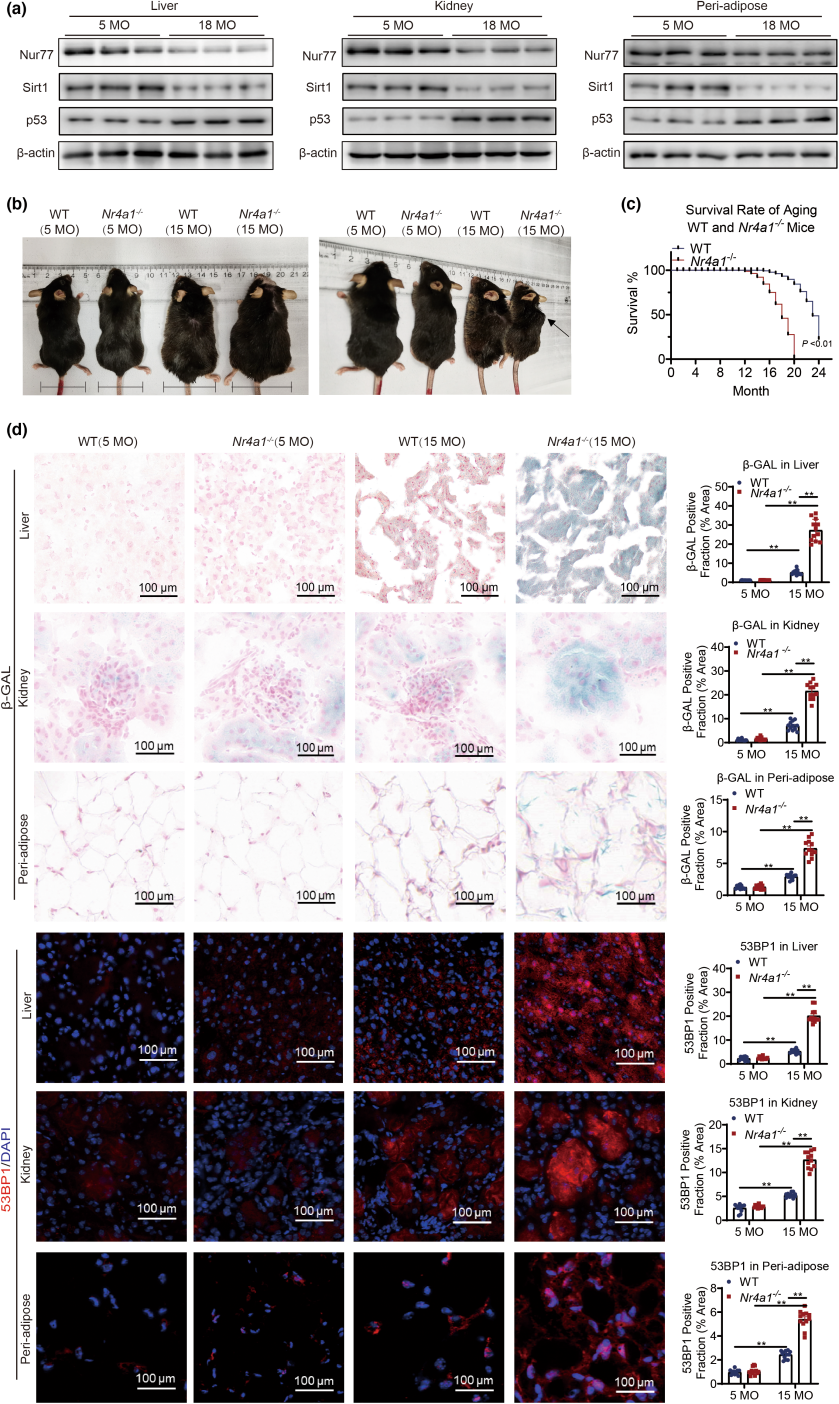
Nur77 deficiency accelerates the aging process in multiple organs. (a) Expression of Nur77, Sirt1, and p53 in the liver, kidney, and peri‐adipose tissue of aged mice. (b) Morphology of 5‐ and 15‐month‐old WT and *Nr4a1* ^−/−^ mice. (c) Survival curves for WT (*n* = 30) and *Nr4a1* ^−/−^ mice (*n* = 30). (d) 53BP1 staining and β‐galactosidase staining in the liver, kidney, and peri‐adipose tissue of 5‐ and 15‐month‐old WT and *Nr4a1* ^−/−^ mice. Scale bar: 100 μm. The data were analyzed by two‐way ANOVA followed by a multiple comparisons test. The results are plotted as the mean ± standard error. **p ≤ 0.01. ATM, ataxia‐telangiectasia mutated protein; Bax, B‐cell lymphoma 2‐associated X‐protein; Chk2, checkpoint kinase 2; β‐GAL, β‐galactosidase; DCFH‐DA, 2′,7′‐dichlorofluorescein diacetate; DNA, deoxyribonucleic acid; γH2AX, phosphorylated histone 2AX; H_2_O_2_, hydrogen peroxide; MEFs, mouse embryonic fibroblasts; NAC, *N*‐acetyl‐l‐cysteine; Nur77, nuclear hormone receptor 77; OS, serum extracted from aged mice; p53, tumor protein p53; Sirt1, sirtuin 1 protein; YS, serum extracted from young mice; WT, wild‐type.

To determine the regulatory mechanism that affects multiple organs during aging, we examined differentially expressed genes in three different naturally aged tissues in NCBI GEO datasets. We detected 461 genes (Figure S1a) that were mainly regulated by transcription factors such as chromodomain‐helicase‐DNA‐binding protein 7 (Chd7), p53, and signal transducer and activator of transcription 3 (Stat3) (Figure S1b). The differentially expressed genes were significantly enriched in the cell‐cycle process (GO:0010564), regulation of cell death (GO:0010942), and stem cell proliferation (GO:0072091) (Figure S1c). Two genes were identified from the intersection of differentially expressed genes in the abovementioned aging‐related signaling pathways: *Igf1r* and *Nr4a1* (Figure S1d). The relationship between insulin‐like growth factor‐1 (Igf)/Igf1r signaling and aging has been extensively studied (Kim & Lee, 2019; Lee & Kim, 2018; Narasimhan et al., 2009). Although the results of different studies have been controversial, inhibiting this signaling pathway has been shown to extend the lifespan of several species. By contrast, *Nr4a1* encodes the Nur77 protein, and its association with longevity and aging is still unknown. We verified Nur77 expression in the aforementioned tissues and several other tissues in naturally aged mice. Nur77 and the longevity protein Sirt1 were significantly decreased, while the aging‐associated protein p53 was significantly increased in aged tissues, including liver, kidney, peri‐adipose, lung, and brain tissue (Figure 1a, Figure S1e–g), suggesting a potential role for Nur77 in the aging process.

To investigate the effects of Nur77 on aging, we established a natural aging model in wild‐type (WT) and *Nr4a1* ^−/−^ mice (Figure S2a). The pathologic phenotypes of 15‐month‐old *Nr4a1* ^−/−^ mice showed more obvious alopecia, intervertebral disk degeneration, and greater abdominal circumferences than WT mice of the same age, which may be due to the role of Nur77 in lipid‐lowering (Figure 1b, Figure S2b). Nur77 deficiency caused a rough decline in the median lifespan compared with that of WT mice (Figure 1c). Impaired glucose tolerance and increased serum lipid levels, including triglycerides (TGs) and total cholesterol (T‐CHO), were more evident in 15‐month‐old *Nr4a1* ^−/−^ mice than in WT mice of the same age (Figure S2c–f). Moreover, senescence‐associated beta‐galactosidase (β‐GAL) staining, p53‐binding protein 1 (53BP1) staining, and dihydroethidium (DHE) staining, which are indicative of accelerated organ aging, the DNA damage response and ROS accumulation, respectively, were observed in multiple organs in *Nr4a1* ^−/−^ mice (15 MO) (Figure 1g,h, Figures S1h–S4). These results indicated that Nur77 was involved in regulating the aging process.

### 2.2 Nur77 attenuates cell senescence by preventing overactivation of the DNA damage response

**FIGURE 2:**
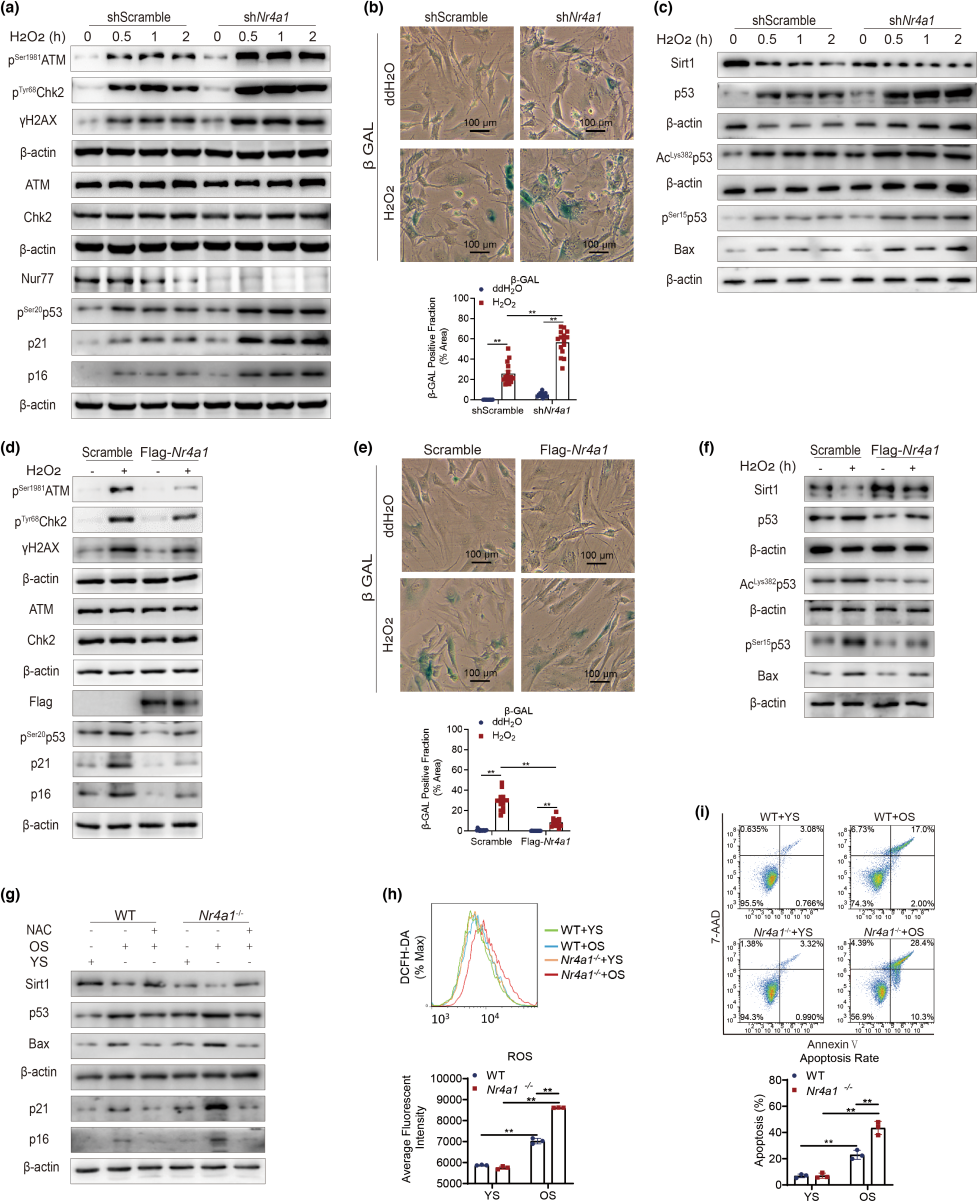
Nur77 attenuates cellular senescence by preventing the overactivation of the DNA damage response. (a) The expression of DNA damage response pathway factors (γH2AX, ATM, Chk2, p53, p21, p16) in shScramble and sh*Nr4a1* HEK‐293T cells under H_2_O_2_ stimulation. *n* = 3 independent experiments. (b) β‐Galactosidase staining of shScramble and sh*Nr4a1* HEK‐293T cells under H_2_O_2_ stimulation. (c) The expression of Sirt1, p53, and Bax in shScramble and sh*Nr4a1* HEK‐293T cells under H_2_O_2_ stimulation. *n* = 3 independent experiments. (d) The expression of DNA damage response pathway factors (γH2AX, ATM, Chk2, p53, p21, p16) in Scramble and Flag‐*Nr4a1* HEK‐293T cells under H_2_O_2_ stimulation. *n* = 3 independent experiments. (e) β‐Galactosidase staining of Scramble and Flag‐*Nr4a1* HEK‐293T cells under H_2_O_2_ stimulation. (f) The expression of Sirt1, p53, and Bax in Scramble and Flag‐*Nr4a1* HEK‐293T cells under H_2_O_2_ stimulation. *n* = 3 independent experiments. (g) The expression of Sirt1, p53, Bax, p21, and p16 in WT and *Nr4a1* ^−/−^ mouse embryonic fibroblasts (MEFs) treated with serum from aged mice (OS) or young mice (YS) and *N*‐acetyl‐l‐cysteine (NAC). *n* = 2 independent experiments. (h) Reactive oxygen species (ROS) levels in WT and *Nr4a1* ^−/−^ MEFs treated with serum from OS or YS and analyzed by flow cytometry. *n* = 3 independent experiments. (i) The apoptosis rates of WT and *Nr4a1* ^−/−^ MEFs treated with serum from OS or YS and analyzed by flow cytometry. *n* = 3 independent experiments. The data were analyzed by two‐way ANOVA followed by a multiple comparisons test. The results are plotted as the mean ± standard deviation (*n* ≤ 6) or standard error (*n* > 6). **p ≤ 0.01. β‐GAL, β‐galactosidase; DCFH‐DA, 2′,7′‐dichlorofluorescin diacetate; NAC, *N*‐acetyl‐l‐cysteine; OS, serum extracted from aged mice; YS, serum extracted from young mice.

Oxidative stress is an upstream pathogenic event that leads to cell senescence and tissue aging. Since mice lacking Nur77 showed elevated ROS levels and DNA damage in vivo, we tested the effect of Nur77 on cell senescence via the DNA damage response. *Nr4a1* knockdown increased the hydrogen peroxide (H_2_O_2_)‐induced sustained DNA damage response in HEK‐293T cells, as indicated by the changes in the phosphorylation states of histone variant H2AX at Ser139 (γH2AX), ataxia‐telangiectasia mutated (ATM) kinase at Ser1981 and checkpoint kinase 2 (Chk2) at Tyr68 (Figure 2a). Moreover, *Nr4a1*‐knockdown HEK‐293T cells exhibited more severe cell senescence phenotypes, including increased expression of β‐GAL staining and levels of cyclin‐dependent kinase inhibitors p21 and p16, than shScramble cells in response to H_2_O_2_ stimulation (Figure 2a,b). The expression of Sirt1 was decreased in the sh*Nr4a1* group, whereas the acetylation state of p53 at Lys382 (deacetylation site of p53 by Sirt1), the phosphorylation state of p53 at Ser15 and its downstream protein B‐cell lymphoma 2‐associated X‐protein (Bax) were all increased when *Nr4a1* was knocked down (Figure 2c). By contrast, *Nr4a1* overexpression decreased the DNA damage response and cell senescence and elevated the expression of Sirt1 (Figure 2d–f).

We also performed relevant verifications in primary cells. We used extracted serum from old mice and young mice and administered it to mouse embryonic fibroblasts (MEFs) extracted from WT and *Nr4a1* ^−/−^ mouse embryos. Similar to the abovementioned results, *Nr4a1* ^−/−^ MEFs exhibited more significant increases in p53, p21, p16, Bax, ROS, and cell apoptosis and a decrease in Sirt1 when stimulated with serum from old mice (OS) than WT MEFs (Figure 2g–i). When MEFs were treated with the antioxidants NAC and OS, no obvious alterations in p53, p21, p16, Bax, or Sirt1 were observed in the presence of serum from young mice (YS) (Figure 2g). These results showed that Nur77 attenuates cell senescence by preventing overactivation of the oxidative stress‐induced DNA damage response.

### 2.3 Nur77 attenuates oxidative stress‐induced cell senescence by enhancing Sirt1 homeostasis

**FIGURE 3:**
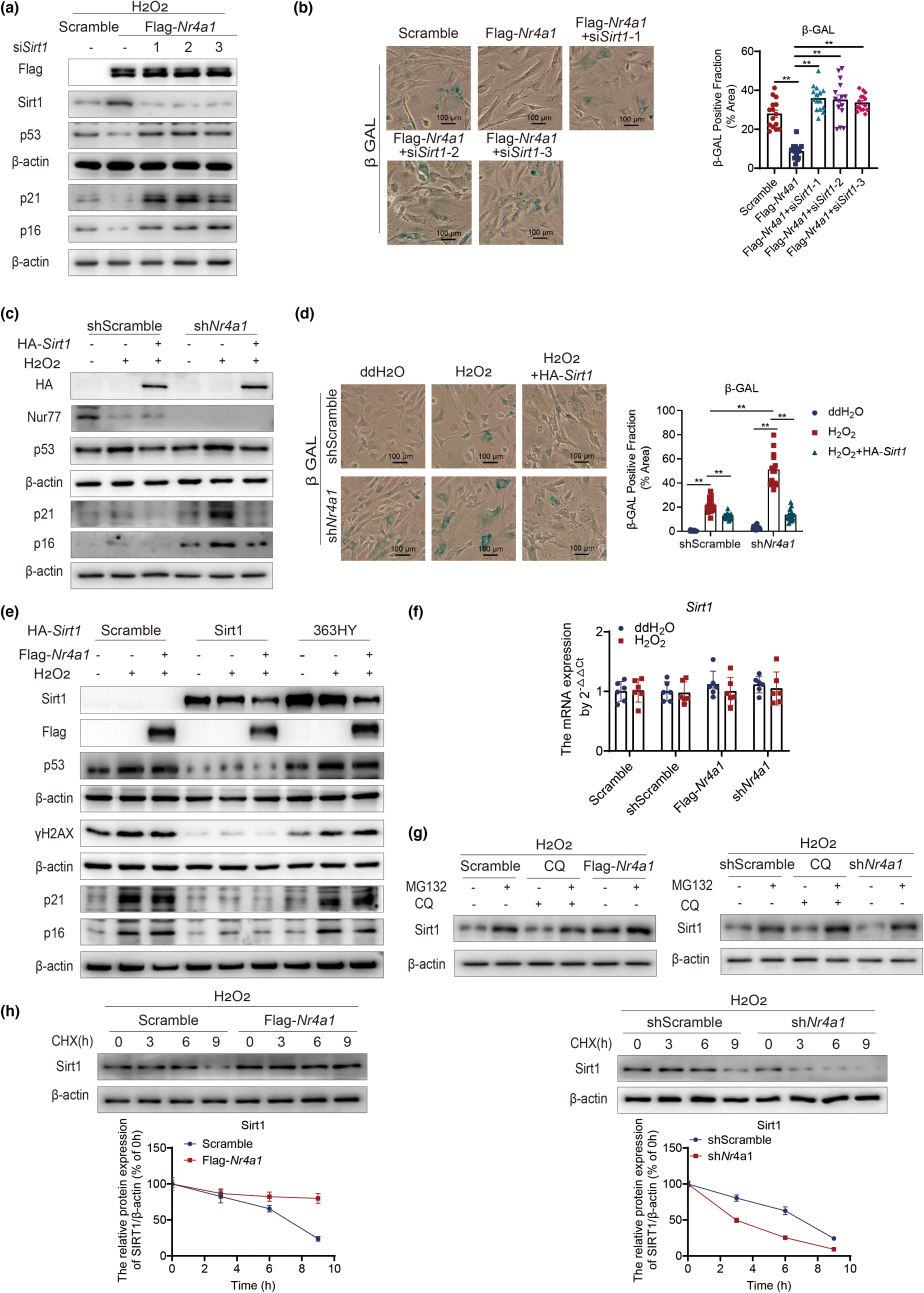
Nur77 attenuates oxidative stress‐induced cell senescence by enhancing the homeostasis of Sirt1. (a) The effects of Nur77 on p53, p21, and p16 in the presence or absence of Sirt1 in HEK‐293T cells under H_2_O_2_ stimulation. *n* = 3 independent experiments. (b) β‐Galactosidase staining of Flag‐*Nr4a1* HEK‐293T cells in the presence or absence of Sirt1 under H_2_O_2_ stimulation. (c) The effects of Sirt1 overexpression on p53, p21, and p16 in sh*Nr4a1* HEK‐293T cells under H_2_O_2_ stimulation. *n* = 3 independent experiments. (d) β‐Galactosidase staining of sh*Nr4a1* HEK‐293T cells overexpressing Sirt1 under H_2_O_2_ stimulation. (e) p53, p21, p16, and γh2AX expression levels in *Sirt1* ^−/−^ HEK‐293T cells with or without Nur77 overexpression and rescued with WT or 363HY *Sirt1*. *n* = 2 independent experiments. (f) *Sirt1* mRNA expression in the presence or absence of Nur77. *n* = 6 independent experiments. (g) Sirt1 levels in the presence or absence of Nur77 and 50 μM Z–Leu–Leu–Leu–al (MG132) for 4 h or 20 μM chloroquine for 10 h. *n* = 3 independent experiments. (h) Sirt1 expression in the presence or absence of Nur77 and 100 μg/mL cycloheximide. *n* = 3 independent experiments. The data were analyzed by two‐way ANOVA followed by a multiple comparisons test. The results are plotted as the mean ± standard deviation (*n* ≤ 6) or standard error (*n* > 6). **p ≤ 0.01. β‐GAL, β‐galactosidase; CHX, cycloheximide; CQ, chloroquine; γH2AX, phosphorylated histone 2AX; H_2_O_2_, hydrogen peroxide; MG132, carbobenzoxy–Leu–Leu–leucinal; mRNA, messenger ribonucleic acid; Nur77, nuclear hormone receptor 77; p53, tumor protein p53; Sirt1, sirtuin 1 protein; MG132, Z–Leu–Leu–Leu–al; WCL, whole cell lysate.

The orphan nuclear receptor Nur77 (also called TR3) shares common regulatory signaling pathways with Sirt1, including metabolic homeostasis, ROS removal, and a decline in the inflammatory response (Hedrick et al., 2019; Hu et al., 2017; Li et al., 2018; Yang et al., 2020). We therefore hypothesized that Nur77 could attenuate oxidative stress‐induced cell senescence via Sirt1. Sirt1 expression was silenced by transfecting *Sirt1* siRNA in *Nr4a1*‐overexpressing HEK‐293T cells. The inhibitory effect of Nur77 on cell senescence‐related indicators (including p53, p21, p16, and β‐GAL staining) was significantly offset when Sirt1 expression was silenced in the context of H_2_O_2_ stimulation (Figure 3a,b). The overexpression of Sirt1 in sh*Nr4a1* HEK‐293T cells resulted in a significant decline in cell senescence‐related indicators under H_2_O_2_ stimulation (Figure 3c,d). Therefore, the inhibitory effect of Nur77 on cell senescence requires Sirt1.

We further investigated whether the inhibitory effect of Nur77 on cell senescence was dependent on the deacetylation function of Sirt1. We administered wild‐type (WT) *Sirt1* and deacetylase‐inactive mutant (363HY) *Sirt1* plasmids to *Sirt1* ^−/−^ HEK‐293T cells and examined DNA damage response (rH2AX) and cell senescence‐related indicators (p53, p21, and p16). In *Sirt1* ^−/−^ HEK‐293T cells, Nur77 overexpression attenuated the DNA damage response and cell senescence, as indicated by changes in γh2AX, p53, p21, and p16, when rescued with WT *Sirt1* but not when rescued with 363HY *Sirt1* (Figure 3e). These results suggested that the improvement in cell senescence mediated by Nur77 is partly dependent on the deacetylase function of Sirt1.

Next, we explored the regulatory effect of Nur77 on Sirt1. As Nur77 belongs to the nuclear receptor family, we first tested whether Nur77 was involved in the transcriptional regulation of Sirt1. The levels of *Sirt1* mRNA showed no obvious alterations in the presence or absence of Nur77 (Figure 2f). We then tested whether Nur77 inhibited Sirt1 protein degradation by administering MG132 or chloroquine (CQ) to HEK‐293T cells with or without *Nr4a1*. MG132 treatment restored Sirt1 protein expression in H_2_O_2_‐induced senescent cells, including *Nr4a1*‐overexpressing and *Nr4a1*‐knockdown senescent cells (Figure 3g). However, treatment with the autophagy inhibitor CQ alone failed to rescue the change in Sirt1 protein levels in senescent cells, but when combined with MG132, it restored Sirt1 expression (Figure 3g). The overexpression of Nur77 prolonged the half‐life of Sirt1 in cells treated with the protein synthesis inhibitor cycloheximide (CHX), whereas Nur77 knockdown significantly shortened the half‐life (Figure 3h). This result indicated that Nur77 contributed to Sirt1 stability by inhibiting its proteasomal degradation.

### 2.4 Nur77 enhances Sirt1 homeostasis via negative transcriptional regulation of MDM2

**FIGURE 4:**
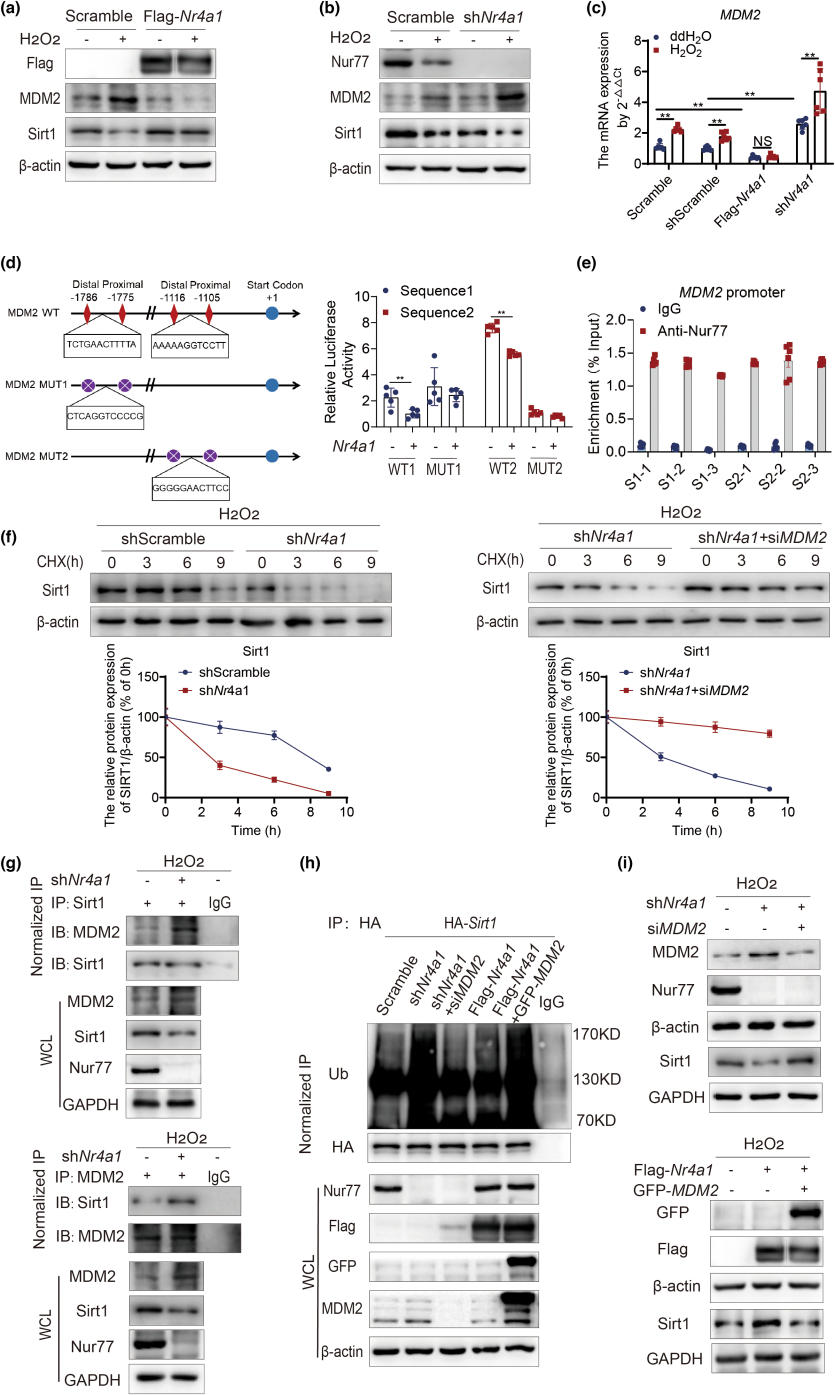
Nur77 enhances Sirt1 homeostasis via negative transcriptional regulation of MDM2. (a) The expression of MDM2 and Sirt1 in Scramble and Flag‐*Nr4a1* HEK‐293T cells under H_2_O_2_ stimulation. *n* = 3 independent experiments. (b) The expression of MDM2 and Sirt1 in shScramble and sh*Nr4a1* HEK‐293T cells under H_2_O_2_ stimulation. *n* = 3 independent experiments. (c) *MDM2* mRNA expression in the presence or absence of Nur77. *n* = 6 independent experiments. (d) Dual‐luciferase reporter assay results of HEK‐293T cells expressing Nur77 and a luciferase reporter containing wild‐type (WT) or mutant (MUT) *MDM2*. *n* = 5 independent experiments. (e) Chromatin immunoprecipitation (ChIP) assays performed on HEK‐293T cells with an antibody against Nur77 or IgG as a control. *n* = 5 independent experiments. (f) The effects of silencing *MDM2* on Sirt1 expression in sh*Nr4a1* HEK‐293T cells treated with 100 μg/mL cycloheximide. *n* = 3 independent experiments. (g) Co‐IP analysis of the interaction between MDM2 and Sirt1 in sh*Nr4a1* HEK‐293T cells under H_2_O_2_ stimulation. *n* = 2 independent experiments. (h) Analysis of the ubiquitination of Sirt1 in the presence or absence of Nur77 or MDM2. *n* = 2 independent experiments. (i) Sirt1 expression in the presence or absence of Nur77 or MDM2. *n* = 2 independent experiments. The data were analyzed by two‐way ANOVA followed by a multiple comparisons test. The results are plotted as the mean ± standard deviation (*n* ≤ 6) or standard error (*n* > 6). **p ≤ 0.01. ChIP, chromatin immunoprecipitation; CHX, cycloheximide; Co‐IP, co‐immunoprecipitation; GAPDH, glyceraldehyde 3‐phosphate dehydrogenase; GFP, green fluorescent protein; HA, human influenza hemagglutinin; H_2_O_2_, hydrogen peroxide; IB, immunoblot; IgG, immunoglobin G; IP, immunoprecipitation; MDM2, murine double minute 2 protein; mRNA, messenger ribonucleic acid; MUT, mutant; Nur77, nuclear hormone receptor 77; Sirt1, sirtuin 2 protein; Ub, ubiquitin; WCL, whole cell lysate.

We then investigated the mechanism by which Nur77 enhances the homeostasis of the Sirt1 protein. Along with Sirt1, Nur77 belongs to the group of nuclear and cytoplasmic shuttling proteins. However, we found no evidence of an interaction between Nur77 and the Sirt1 protein, suggesting that Nur77 did not stabilize Sirt1 in a direct manner (Figure S5). We further investigated whether Nur77 regulates the proteasomal degradation of Sirt1 through its E3 ligase. We reanalyzed the renal transcriptome data of *Nr4a1* ^−/−^ rats in NCBI GEO datasets and found that the top pathways associated with differentially expressed gene enrichment included the proteasome pathway (GO: 0043161) and apoptosis pathway (GO: 0097190) (Figure S6a). The E3 ligase MDM2 was one of the differentially expressed genes in these two pathways (Figure S6b). In addition, MDM2 ranked second among the major E3 ligases predicted by the UbiBrowser website for Sirt1 (Figure S6c). Indeed, we found that Nur77 negatively regulated the expression of MDM2 at both the mRNA and protein levels (Figure 4a–c). We used the Jasper website to predict whether Nur77 could regulate MDM2 transcription, and the results showed that there were 16 Nur77‐binding regions upstream of the MDM2 promoter (Table S1). Two highly rated binding sequences were selected and verified by luciferase reporter and chromatin immunoprecipitation (ChIP) assays (Figure 4d). The results of the luciferase reporter assay showed that Nur77 could negatively regulate *MDM2* transcription (Figure 4d). The ChIP results showed that the promoter of MDM2 was effectively enriched by the Nur77 antibody compared with the IgG control in the two binding regions (Figure 4e). These results demonstrated that Nur77 could inhibit *MDM2* transcription by binding to its promoter.

To determine whether Nur77 deficiency could accelerate Sirt1 degradation by activating MDM2, we first observed the change in the half‐life of Sirt1 when *MDM2* was knocked down in sh*Nr4a1* HEK‐293T cells. Knockdown of *MDM2* prolonged the half‐life of the Sirt1 protein in sh*Nr4a1* HEK‐293T cells stimulated with H_2_O_2_ (Figure 4f). Immunoprecipitation showed that MDM2 binding to Sirt1 was significantly increased in sh*Nr4a1* HEK‐293T cells (Figure 4g). Moreover, *Nr4a1* knockdown increased the ubiquitination of Sirt1 and decreased its expression, which could be rescued by silencing *MDM2* (Figure 4h,i). Consistently, Nur77 overexpression protected Sirt1 from ubiquitination and increased its expression, which could be diminished by overexpressing *MDM2* (Figure 4h,i). These results indicate that Nur77 enhances the stabilization of the Sirt1 protein by suppressing MDM2 during oxidative stress‐induced cell senescence.

### 2.5 Nur77 deficiency stabilizes p53 protein expression in response to oxidative stress

**FIGURE 5:**
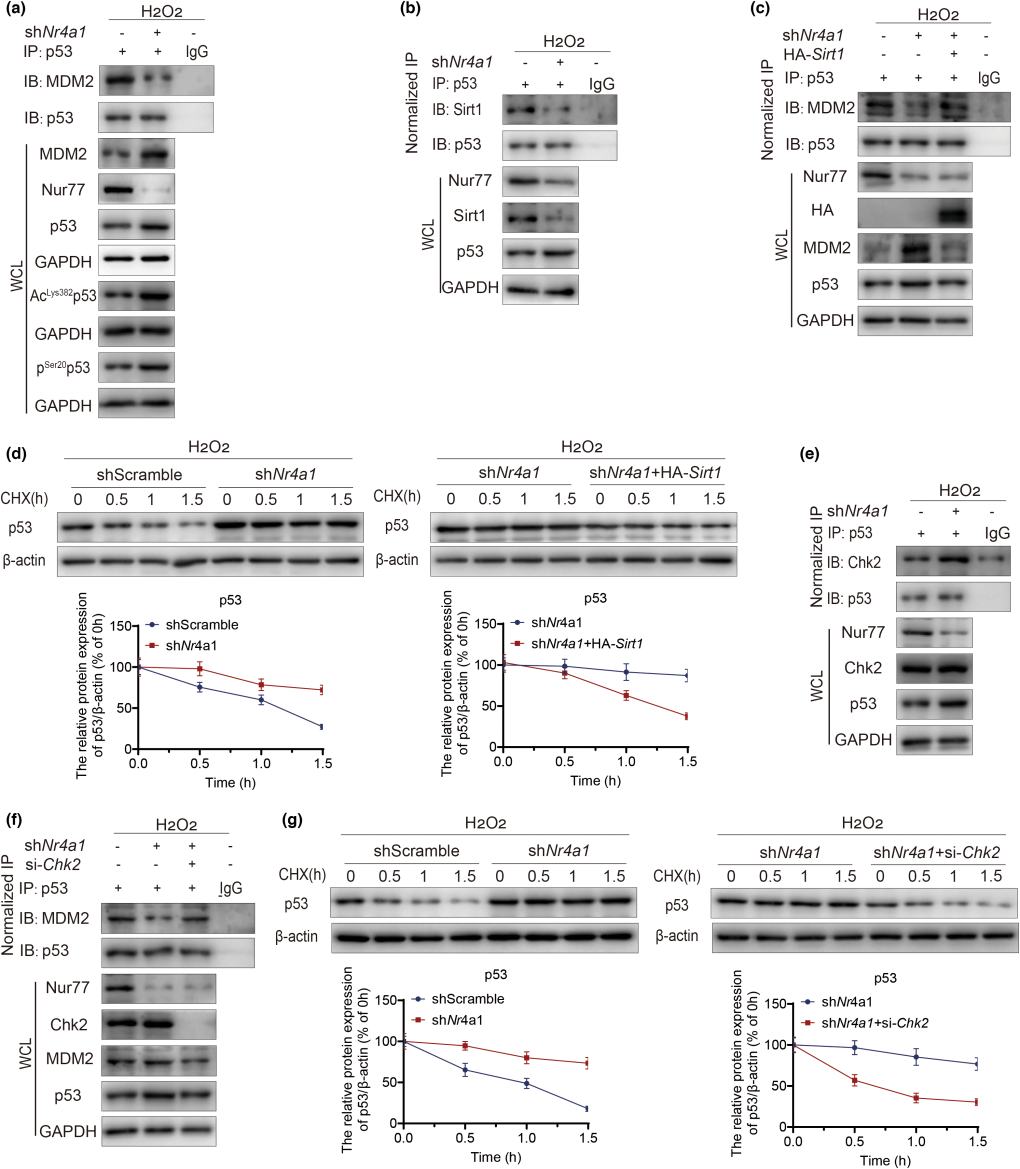
Nur77 deficiency stabilizes p53 protein expression in response to oxidative stress. (a) Co‐IP analysis of the interaction between MDM2 and p53 in sh*Nr4a1* HEK‐293T cells under H_2_O_2_ stimulation. *n* = 2 independent experiments. (b) Co‐IP analysis of the interaction between p53 and Sirt1 in sh*Nr4a1* HEK‐293T cells under H_2_O_2_ stimulation. *n* = 2 independent experiments. (c) Co‐IP analysis of the interaction between MDM2 and p53 in sh*Nr4a1* HEK‐293T cells with or without *Sirt1* overexpression under H_2_O_2_ stimulation. *n* = 2 independent experiments. (d) The effects of *Sirt1* overexpression on p53 expression in sh*Nr4a1* HEK‐293T cells treated with 100 μg/mL cycloheximide. *n* = 2 independent experiments. (e) Co‐IP analysis of the interaction between p53 and Chk2 in sh*Nr4a1* HEK‐293T cells under H_2_O_2_ stimulation. *n* = 2 independent experiments. (f) Co‐IP analysis of the interaction between MDM2 and p53 in sh*Nr4a1* HEK‐293T cells with or without *Chk2* silencing under H_2_O_2_ stimulation. *n* = 2 independent experiments. (g) The effects of silencing *Chk2* on p53 expression in sh*Nr4a1* HEK‐293T cells treated with 100 μg/mL cycloheximide. *n* = 2 independent experiments. CHX, cycloheximide; Chk2, checkpoint kinase 2; Co‐IP, co‐immunoprecipitation; GAPDH, glyceraldehyde 3‐phosphate dehydrogenase; HA, human influenza hemagglutinin; H_2_O_2_, hydrogen peroxide; IB, immunoblot; IP, immunoprecipitation; MDM2, murine double minute 2 protein; Nur77, nuclear hormone receptor 77; Sirt1, sirtuin 2 protein; WCL, whole cell lysate.

The MDM2 is a classical E3 ligase of p53. We further investigated whether Nur77 deficiency affected p53 expression in response to oxidative stress. Enhanced acetylation of p53 is widely believed to be closely related to its stability and activation in response to cellular stress (Barlev et al., 2001; Knights et al., 2006; Luo et al., 2000; Zhao et al., 2008). C‐terminal acetylation‐deficient p53‐6KR knock‐in mice have reduced p53‐dependent gene expression after DNA damage (Feng et al., 2005). Moreover, DNA damage‐induced phosphorylation of p53 by the kinases ATM/Chk2 at Ser20 disrupts the p53‐MDM2 interaction, which stabilizes p53 for activation (Kruse & Gu, 2009). Knockdown of *Nr4a1* decreased the interaction of p53 and MDM2 after H_2_O_2_ stimulation, accompanied by an increase in the acetylation of p53 at Lys382 and the phosphorylation of p53 at Ser20 (Figure 5a). Nur77 deficiency decreased Sirt1 expression and reduced p53‐Sirt1 binding, which was responsible for the deacetylation of p53 at Lys382 (Figure 5b). Sirt1 overexpression increased MDM2 binding to p53 in *Nr4a1*‐knockdown HEK‐293T cells (Figure 5c), which shortened the half‐life of p53 (Figure 5d). Nur77 deficiency further activated the ATM/Chk2 pathway, resulting in increased phosphorylation of p53 at Ser20. We transfected NCI‐H1299 cells, a natural p53‐null cell line, with WT p53, S20A p53 (nonphosphorylation), or S20E p53 (phosphorylation mimic) (Figure S7). We found that *Nr4a1* knockdown increased the expression of γH2AX, p21, and p16 in WT and S20E‐transfected cells but not in S20A‐transfected cells (Figure S7). This finding suggested that the activation and stabilization of p53 in sh*Nr4a1* HEK‐293T cells were partially dependent on its phosphorylation at Ser20. We also examined whether the increase in p53 stability when *Nr4a1* was knocked down was dependent on the ATM/Chk2 pathway. Nur77 deficiency increased p53‐Chk2 binding, which was responsible for the phosphorylation of p53 at Ser20 (Figure 5e). Silencing *Chk2* increased MDM2 binding to p53 in *Nr4a1*‐knockdown HEK‐293T cells (Figure 5f), which shortened the half‐life of p53 (Figure 5g). These results showed that Nur77 deficiency could stimulate an increase in p53 acetylation at Lys382 and phosphorylation at Ser20 by downregulating Sirt1 and activating the DNA damage response, thus stabilizing p53 expression.

### 2.6 Nur77 deficiency accelerates podocyte senescence and kidney function decline

**FIGURE 6:**
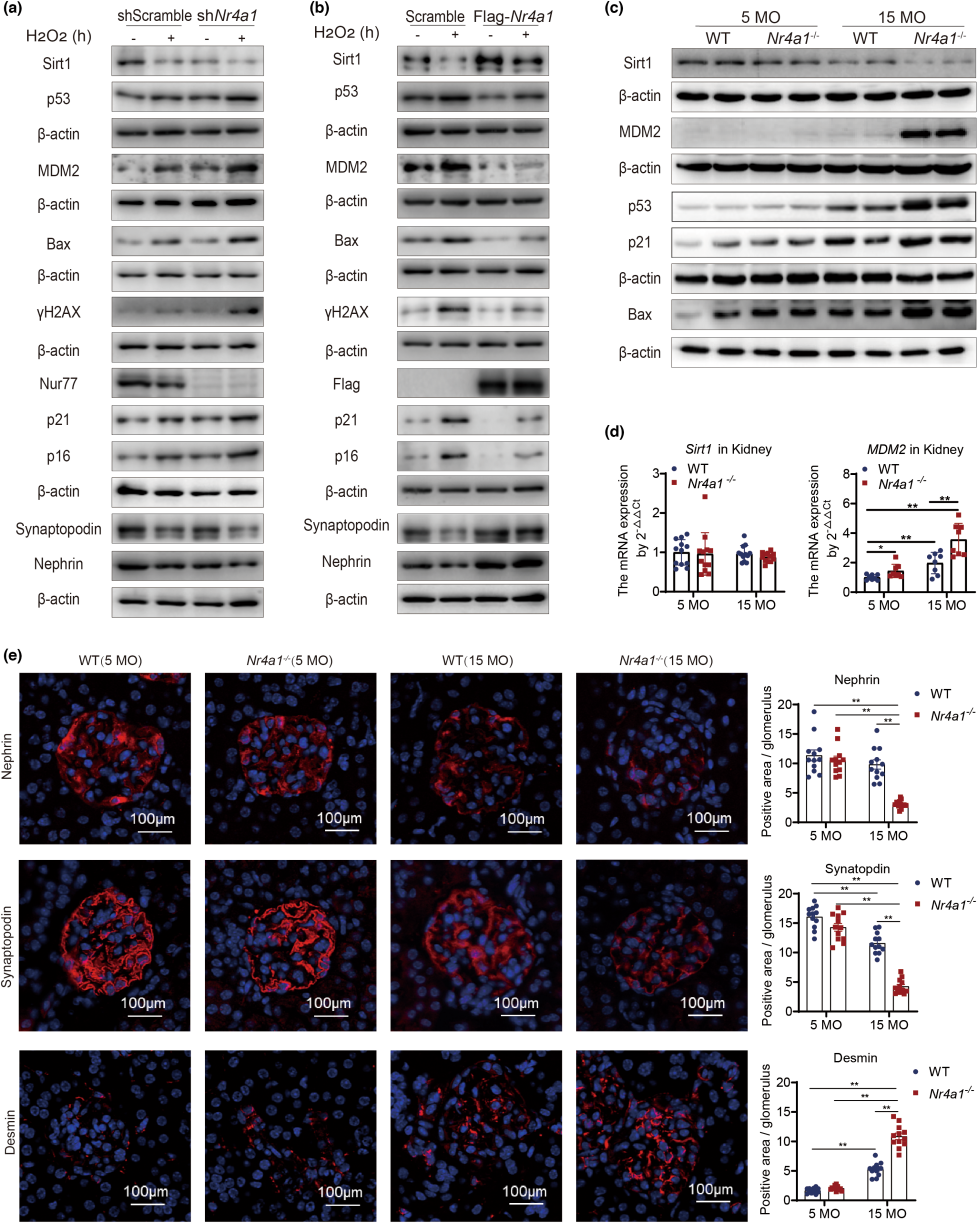
Nur77 deficiency accelerates podocyte senescence and glomerular damage. (a) The expression of Sirt1, MDM2, γH2AX, p53, p21, p16, and podocyte markers (nephrin, synaptopodin) in shScramble and sh*Nr4a1* podocytes under H_2_O_2_ stimulation. *n* = 2 independent experiments. (b) The expression of Sirt1, MDM2, γH2AX, p53, p21, p16, and podocyte markers (nephrin, synaptopodin) in scramble and Flag‐*Nr4a1* podocytes under H_2_O_2_ stimulation. *n* = 2 independent experiments. (c) The expression of Sirt1, MDM2, p53, p21, and Bax in kidney tissue of 5‐ and 15‐month‐old WT and *Nr4a1* ^−/−^ mice. *n* = 2 independent experiments. (d) *Sirt1* and *MDM2* mRNA expression in kidney tissue of 5‐ and 15‐month‐old WT and *Nr4a1* ^−/−^ mice. *n* = 6 independent experiments. (e) Immunofluorescence staining of nephrin, synaptopodin, and desmin in the glomeruli of 5‐ and 15‐month‐old WT and *Nr4a1* ^−/−^ mice. The data were analyzed by two‐way ANOVA followed by a multiple comparisons test. The results are plotted as the mean ± standard error. **p ≤ 0.01. MO, month‐old.

To gain functional insight, we investigated the effects of Nur77 deficiency on the kidney during the aging process and compared the changes in Sirt1, MDM2, and the DNA damage response pathway in the presence or absence of Nur77. Similar to its expression in HEK‐293T cells, *Nr4a1* deficiency in podocytes increased MDM2, decreased Sirt1, and hyperactivated the ATM/Chk2/p53/p21/p16 pathway, ultimately leading to a reduction in podocyte‐associated markers, including nephrin and synaptopodin (Figure 6a). By contrast, *Nr4a1* overexpression stabilized the expression of Sirt1 and inhibited the expression of cell senescence‐related markers, including p21 and p16 (Figure 6b). We found that the protein expression of Sirt1 in the kidney was significantly reduced with age and observed an additional 39% decrease in the kidney with Nur77 deficiency compared with that in WT mice at 15 months of age (Figure 6c). Contrary to the decreased expression of Sirt1 that occurred in the presence of Nur77 deficiency at 15 months of age, the protein expression of MDM2 was significantly increased (Figure 6c). Consequently, both conditions increased the level of p53/p21/Bax (Figure 6c). Consistent with the in vitro results, *Sirt1* mRNA levels were not altered in the kidney, regardless of the presence or absence of Nur77, whereas the mRNA expression of *MDM2* increased with Nur77 deficiency (Figure 6d). Additionally, Nur77 deficiency was associated with decreased expression of podocyte‐associated markers, including nephrin and synaptopodin, and increased expression of the podocyte injury marker desmin in the glomeruli of 15‐month‐old mice (Figure 6e). As such, Nur77 regulates the same biological mechanisms during the aging process of podocytes and the kidney as it does during oxidative stress‐induced cell senescence.

To evaluate the effect of Nur77 on kidney function, serum creatinine (SCr) and blood urea nitrogen (BUN) levels, urine volume (UV), the creatinine clearance rate (CCr), 24 h urinary albumin (ALB), and the ratio of ALB to urinary creatinine (ACr) were analyzed (Figure S8a–f). Nur77 deficiency increased SCr, BUN, ALB, and ACr levels, whereas UV and the CCr were markedly decreased in 15‐month‐old mice. These results suggest that Nur77 deficiency aggravates the impairment of renal function during the aging process. Morphological analysis of 15‐month‐old mice revealed that Nur77 deficiency aggravated glomerular and podocyte injuries, as determined by periodic acid–Schiff (PAS) staining and transmission electron microscopy (TEM). Thickening of Bowman's capsule, and noticeable intestinal and periglomerular fibrosis were all observed in 15‐month‐old kidneys in which *Nr4a1* was mutated (Figures S8g–j and S9). Nur77 deficiency in mice also increased podocyte apoptosis, as determined by TdT‐mediated dUTP nick end labeling (TUNEL) staining (Figure S8g). Nur77 deficiency aggravated podocyte senescence and apoptosis in the kidney, and Nur77 expression in renal compartments exhibited dichotomous associations with aging nephropathy.

### 2.7 Resveratrol rescues Nur77 deficiency‐induced kidney damage in aged mice

To identify whether Nur77 deficiency–mediated Sirt1 degradation was the key process in aging nephropathy, we treated aged WT and *Nr4a1* ^−/−^mice with the activator, resveratrol (Res). After being treated with 400 mg/kg resveratrol for 3 months, impaired glucose tolerance, and increased serum FBG and lipid levels, including TGs and T‐CHO in aged WT and *Nr4a1* ^−/−^mice were all alleviated (Figure S10a–e). Moreover, activation of Sirt1 restored the Nur77 deficiency‐induced decrease in renal function. Res treatment decreased SCr, BUN, ALB, and ACr levels, and increased UV and CCr in both aged WT and *Nr4a1* ^−/−^mice (Figure S10f–k). Morphological analysis revealed that Res treatment improved glomerular and podocyte injuries, as determined by thinned GBM, decreased β‐GAL staining, decreased podocyte injury marker desmin, and elevated podocyte‐associated markers (nephrin and synaptopodin) (Figure S10g). These results suggest that supplementing or activating Sirt1 helped improve Nur77 deficiency‐aggravated aging nephropathy.

## 3 DISCUSSION

The classic longevity protein Sirt1 gradually decreases during the natural aging process, but the underlying regulatory mechanism remains unclear. Our findings revealed that the loss of Nur77 with age augmented the DNA damage response and cell senescence and accelerated the aging process in several mouse tissues. We revealed that Nur77 stabilized the Sirt1 protein by reducing its degradation by the proteasome through negative transcriptional regulation of its E3 ligase MDM2. In addition, we confirmed the compelling role of Nur77 in protecting against aging nephropathy via Sirt1. However, these findings need to be validated in other aging diseases to clarify the general role of Nur77‐stabilized Sirt1 against aging.

Nur77 is a nuclear receptor and a negative regulator of inflammatory factor production and fatty acid synthesis (Hedrick et al., 2019; Hu et al., 2017; Li et al., 2018; Yang et al., 2020). In addition, Nur77 has been reported to protect TPβ (a key rate‐limiting enzyme for fatty acid oxidation) against oxidation, thereby maintaining normal fatty acid metabolic processes and preventing an increase in ROS (Li et al., 2018). Although Nur77 can regulate a variety of aging‐related stimuli, the relationship between Nur77 and aging has not been revealed. We found that the Nur77 protein was reduced in multiple aged tissues and cells with oxidative stress‐induced senescence. Nur77 deficiency resulted in ROS accumulation, an enhanced DNA damage response, and increased the senescence markers β‐galactosidase, p53, p21, and p16 in multiple tissues. These phenotypes are consistent with the regulatory role of Nur77 in oxidative stress and lipid metabolism. Fifteen‐month‐old *Nr4a1* ^−/−^ mice exhibited more obvious alopecia, intervertebral disk degeneration, and greater abdominal circumference than WT mice. Mice lacking *Nr4a1* also had significantly shorter lifespans. Accordingly, Nur77 deficiency that occurs with age accelerates the aging process.

Sirt1 is involved in the regulation of various important senescence‐related biological processes, including inhibiting inflammation, the DNA damage response, and cellular apoptosis (Bi et al., 2019; Gomes et al., 2013; Wellman et al., 2017). It is important to clarify the mechanism of the age‐related decline in Sirt1 to delay the aging process. Recent studies have shown no obvious alterations in *Sirt1* mRNA in DNA damage‐induced senescence (Xu et al., 2020). Nur77 and Sirt1 share many similarities in the regulation of aging‐related biological pathways. We found that Nur77 ameliorated the DNA damage response and cell senescence via the deacetylation function of Sirt1. Similar to previous results, our results showed that Nur77 did not affect *Sirt1* mRNA expression under oxidative stress. Our findings suggested that Nur77 indirectly protected the Sirt1 protein against proteasomal degradation by regulating its E3 ligase MDM2. Wu et al. (Zhao, Chen, et al., 2006) presented evidence that Nur77 inhibited MDM2 expression at the mRNA level. Our results further confirmed that Nur77 could bind directly to the promoter of *MDM2* and suppress its transcription. Silencing *MDM2* reversed the decrease in Sirt1 expression, the increase in the DNA damage response, and the cell senescence markers p21 and p16 caused by the decline in Nur77 under stress conditions. It has been suggested that Nur77 downregulates p53 transcriptional activity by blocking its acetylation (Zhao, Chen, et al., 2006). Based on our findings, we hypothesized that this might be related to Nur77‐mediated stabilization of Sirt1 expression, which affects the transcriptional activity of p53.

It is well known that MDM2 is the primary E3 ligase of p53 (Haupt et al., 1997; Peng et al., 2015). In the present study, the activation of MDM2 by Nur77 deficiency selectively degraded Sirt1 but not p53 under oxidative stress. The enhancement of p53 acetylation and phosphorylation levels strongly correlates with protein stabilization and activation in response to cellular stress. Mutations of lysine residues 370, 372, 373, 381, 382, and 386 to arginine residues in p53 (6KR p53 mutant) result in resistance to MDM2‐induced degradation (Rodriguez et al., 2000). Among them, the acetylation of p53 at lysine 373/382 induces the expression of p21 (Zhao, Lu, et al., 2006). Sirt1‐mediated deacetylation of p53 represses p53‐mediated cell growth arrest and apoptosis in response to DNA damage and oxidative stress (Luo et al., 2001). Therefore, the reduction in Sirt1 caused by Nur77 deficiency with age led to the acetylation of p53 at Lys382, reduced the recognition of p53 by MDM2, and caused an increase in the cell‐cycle arrest protein p21. The phosphorylation of p53 at serines 15, 20, and 37 impairs the binding of p53‐MDM2 to inhibit p53‐dependent transactivation (Shieh et al., 1997). Our results showed that Nur77 deficiency‐induced oxidative stress and the DNA damage response, which in turn stimulated p53 phosphorylation. This also explained why p53 was stable in *Nr4a1*‐deficient tissues and cells in response to oxidative stress. These events constituted a positive feedback mechanism that accelerates the aging process: the oxidative stress‐induced reduction in Nur77 disrupts the homeostasis of the Sirt1 protein through MDM2, resulting in cell senescence and reactive oxygen production, which in turn further decreases the expression of Nur77 and accelerates the aging process.

Sirt1 is widely expressed in tubular cells and podocytes in the kidney (Zhong et al., 2018). Podocytes maintain the glomerular filtration barrier, and initial glomerular injury affects podocytes, which are important target cells for the progression of aging nephropathy (Nagata, 2016). Podocyte‐specific deletion of *Sirt1* results in ROS accumulation, inflammatory response, and podocyte loss in the kidney (Chuang et al., 2017; Hong et al., 2018). We further examined the effects of Nur77 in renal aging, which may be representative of the general role of Nur77 in aging diseases. Our results revealed that the expression of Nur77 in the kidney decreased gradually during the natural aging process. Sirt1 was significantly reduced in the kidneys in the context of Nur77 deficiency, corresponding to the activation of senescence signals. Nur77 deficiency aggravated aging‐related morphological changes and functional damage to the kidney. Activating Sirt1 or Nur77 helped improve Nur77 deficiency‐aggravated aging nephropathy. Therefore, Nur77 may be a new therapeutic target to combat aging‐related nephropathy.

Collectively, our study showed for the first time that Nur77 was an important target in combating the aging process. Nur77 is also an important upstream regulator that maintains Sirt1 protein homeostasis during the aging process. Preventing the reduction in Nur77 with age by pharmacological targeting during late adulthood may be a novel approach for the treatment of aging diseases.

## 4 EXPERIMENTAL PROCEDURES

### 4.1 Mouse experiments

All animal experiments were approved by the Animal Ethics Committee of China Medical University (CMU2019277). Wild‐type (WT) and *Nr4a1*‐targeted mutant (No: 006187; *Nr4a1* ^−/−^) mice with a C57BL/6J background were purchased from The Jackson Laboratory (Bar Harbor, ME, USA). *Nr4a1*‐targeted mutant mice were produced with a neomycin cassette introduced to exon 2 of murine Nur77 to block the transcription of both the DNA‐binding domain (DBD) and ligand‐binding domain (LBD) (Lee et al., 1995). All mice were housed in a temperature‐ and climate‐controlled barrier system (23 ± 2°C and 45%–60% relative humidity, 12 h cycle of light and darkness) and fed regular rodent chow. Naturally aging mice were divided into four groups for analysis (5‐, 8‐, 15‐, 18‐month‐old). Young serum (YS) and old serum (OS) were extracted from 5‐ and 18‐month‐old wild‐type mice, respectively, for subsequent serum pharmacology experiments in mouse embryonic fibroblasts (MEFs). The number of surviving WT (*n* = 30) and *Nr4a1* ^*−/−*^ (*n* = 30) mice was recorded every month for 24 months. To study the effect of Nur77 deficiency on the aging process in mice, the mice were divided into groups for analysis: 5‐month‐old mice (WT‐5 MO; *n* = 6 and *Nr4a1* ^−/−^‐5 MO; *n* = 6) and 15‐month‐old mice (WT‐15 MO; *n* = 6 and *Nr4a1* ^−/−^‐15 MO; *n* = 6). Urine samples were collected by placing each mouse in an individual metabolic cage (Tecniplast) for 24 h. The oral glucose tolerance test (OGTT) was performed at 0, 30, 60, 90, and 120 min using an Accu‐Chek meter (Roche). Fasting blood glucose (FBG), total cholesterol (T‐CHO), triglyceride (TAG), creatine (Cr), and urea nitrogen (BUN) levels were determined according to the test kit manufacturer's instructions (Jiancheng). To investigate the effect of Sirt1 activation on kidney injury in aged *Nr4a1* ^−/−^ mice, the mice were divided into four groups for analysis: WT + PBS: 15‐month‐old WT mice treated with PBS for 3 months (*n* = 6), *Nr4a1* ^−/−^ + PBS: 15‐month‐old *Nr4a1* ^−/−^ mice treated with PBS for 3 months (*n* = 6), WT+ Res: 15‐month‐old mice treated with 400 mg/kg resveratrol for 3 months (*n* = 6), *Nr4a1* ^−/−^ + Res: 15‐month‐old *Nr4a1* ^−/−^ mice treated with 400 mg/kg resveratrol for 3 months (*n* = 6). Urine samples were collected for 24 h. OGTT was performed at 0, 30, 60, 90, and 120 min. FBG, T‐CHO, TAG, Cr, and BUN levels were determined.

### 4.2 Histological staining of tissues and analysis

For hematoxylin and eosin (H&E), Sirius red, Masson's trichrome, and periodic acid–Schiff (PAS) staining, tissues were isolated and fixed overnight at 4°C and treated with 30% sucrose for cryoprotection. Kidney sections were dewaxed with xylene and a series of descending ethanol gradients. Then, the sections were stained with Mayer's H&E (Jiancheng) or Sirius Red F3B and a saturated aqueous solution of picric acid (Solarbio), Masson (Jiancheng) or PAS (Solarbio) and observed with a Nikon Eclipse Ni‐U microscope. For immunohistochemistry (IHC), paraffin sections were stained with anti‐Nephrin (Abcam), assessed by light microscopy, and quantified using ImageJ (Wayne Rasband, US National Institutes of Health). For β‐GAL, TUNEL, and immunofluorescence staining, frozen tissue slices were stained with a β‐GAL kit (Beyotime Biotechnology), TUNEL kit (Beyotime), or anti‐53BP1 (NOVUS), respectively, photographed using an Olympus IX‐71 fluorescence microscope and quantified using ImageJ. For DHE staining, 24 h before being euthanized, the mice received a 200 μL intravenous injection of dihydroethidium (25 mg/kg; Sigma–Aldrich), and the frozen tissue slices were photographed using an Olympus IX‐71 fluorescence microscope and quantified using ImageJ.

### 4.3 Transmission electron microscopy (TEM)

For transmission electron microscopy, sample handling and detection were performed by Wuhan Servicebio Technology. Tissues were collected and fixed with 2.5% glutaraldehyde at 4°C. The sections were washed with PBS and fixed in 1% osmium tetroxide at room temperature for 2 h. The specimens were then dehydrated using a series of ascending ethanol gradients and 100% acetone. After being dehydrated, the sections were embedded in Pon 812 resin overnight at 37°C using acetone as a transitional solvent. The ultrathin sections were stained with 2% saturated uranyl acetate and lead citrate. Glomerular basement membrane (GBM) thickness, foot process width, and the number of foot processes per μm of GBM and TEM images were analyzed using ImageJ.

### 4.4 RNA‐sequencing analysis

The RNA‐sequencing data were downloaded and referenced to the Gene Expression Omnibus under accession numbers GSE54714, GSE6591, GSE8150, and GSE25905. A log fold change (log_2_FC) > 1.5 and an adjusted *p* value < 0.05 were set as the thresholds for the identification of differentially expressed genes. Bioinformatic analyses using Metascape pathway analysis (Tripathi et al., 2015) and Ingenuity Pathways Analysis (Kramer et al., 2014) were carried out to determine molecular functions and upstream signaling pathways.

### 4.5 Cell culture and treatments

Mouse embryonic fibroblasts (MEFs) were derived from E14.5 embryos from *Nr4a1* ^+/−^ C57BL/6J mice and cultured in DMEM with 15% FBS (Gibco), 100 units/mL penicillin, and 10 mg/mL streptomycin. Then, the cells were treated with serum from aged mice (OS) or young mice (YS) as a normal control for 24 h. Human embryonic kidney (HEK)‐293T cells were obtained from the American Type Culture Collection and cultured in DMEM with 10% FBS. HNCI‐H1299 cells, a naturally p53‐null cell line, were obtained from the American Type Culture Collection and cultured in 1640 medium with 10% FBS. Immortalized mouse podocyte MPC‐5 cells were purchased from the Research Facilities of Peking Union Medical College (PUMC) Cell Bank. Undifferentiated MPC‐5 cells were cultured in DMEM (Gibco) with 10 U/mL mouse recombinant interferon‐γ (IFN‐γ; Peprotech Inc.) at 33°C in an incubator with 5% CO_2_. Podocytes were cultured at 37°C for 10–14 days in DMEM without IFN‐γ to induce cell differentiation and maturation. For DNA damage‐induced senescence, HEK‐293T cells were treated with 100 μM H_2_O_2_ for 4 h. Transfection was performed using Lipo3000 (Thermo Fisher Scientific).

### 4.6 Plasmids

*Nr4a1* overexpression and shRNA lentiviruses were purchased from GeneChem (Shanghai, China). HA‐tagged WT or deacetylase‐inactive mutant (363HY) *Sirt1* plasmids were constructed as described previously (Wang et al., 2006). *CBP*, *p300*, *GCN5*, and *PCAF* plasmids were constructed as described previously (Yi et al., 2021). *Sirt1*, *MDM2*, and *CBP* siRNAs were purchased from RiboBio. *GFP‐MDM2*, *ubiquitin* (*Ub*), and site‐directed mutagenesis (WT, S20A, S20E) of Flag*‐p53* plasmids was performed using QuikChange XL (Stratagene) and confirmed by sequencing. *Sirt1* ^−/−^ and *Chk2* ^−/−^ Cas9 HEK‐HEK‐293T cells were constructed and provided by Zhang et al. (2020).

### 4.7 Co‐immunoprecipitation (Co‐IP) and western blot analysis

Co‐IP and western blot analysis were performed as previously described (Fu et al., 2020). The different primary antibodies used are listed in Table S2.

### 4.8 Real‐time polymerase chain reaction (PCR)

RNA was isolated from podocytes using an RNeasy Plus Mini Kit (Qiagen). Copy DNA was prepared using a PrimeScript™ RT reagent kit (TaKaRa) followed by quantitative real‐time PCR using SYBR Green (TaKaRa). Relative quantitation was carried out using 2^−ΔΔCT^. The RT–PCR primers are listed in Table S3.

### 4.9 Protein degradation assays

To determine protein half‐lives, cells were pretreated with 100 μM H_2_O_2_ for 6 h and then incubated with 100 μg/mL cycloheximide (CHX; APExBIO) for 0, 3, 6 and 9 h (Sirt1) or 0, 30, 60, and 90 min (p53), followed by sample harvesting and western blot analysis. To determine whether Sirt1 expression was affected by proteasome‐dependent proteolysis, cells were treated with 50 μM MG132 (Selleck Chemicals) for 4 h with/without 20 μM chloroquine (CQ; Sigma–Aldrich) for 10 h. For ubiquitination assays, cells were co‐transfected with HA‐*Sirt1*, *Ubiquitin* (*Ub*), and GFP‐*MDM2* plasmids. At 30 h post‐transfection, the cells were harvested and subjected to co‐immunoprecipitation (Co‐IP) analysis.

### 4.10 Flow cytometry

Apoptosis assessments were made based on allophycocyanin (APC) Annexin V and propidium iodide (PI) staining (Thermo Fisher). Cells were harvested followed by staining with APC Annexin V and PI according to the manufacturer's instructions. ROS were determined by a fluorometric intracellular ROS Kit (Sigma–Aldrich) according to the manufacturer's instructions. After being stimulated, the cells were incubated in Hank's balanced salt solution containing 5 mM 2′,7′‐dichlorodihydrofluorescein diacetate (DCFH‐DA) for 1 h and analyzed by flow cytometry using a FACSCalibur (Becton Dickinson).

### 4.11 Luciferase reporter assay

HEK‐293T cells were cultured at a density of 2 × 10^4^ cells/well in 96‐well culture plates and transfected with 0.2 μg of the WT or mutant *MDM2* dual‐luciferase reporter construct or were co‐transfected with 0.2 μg of the *Nr4a1* luciferase reporter construct and the internal control vector pRL‐TK (Promega) at a ratio of 20:1 (reporter construct: control vector) using LipofectamineTM 2000 (Invitrogen) according to the manufacturer's instructions. Five hours post‐transfection, the transfection medium was removed and replenished with a medium containing 6 μM curcumin (Sigma–Aldrich) solubilized in 100% dimethylsulfoxide (DMSO) (Sigma–Aldrich). Forty‐eight hours post‐transfection, luciferase activity was measured using the Dual‐Luciferase® Reporter Assay System (Promega). Renilla luciferase activity was normalized to firefly luciferase activity in cells that were transfected with the WT or mutant *MDM2* dual‐luciferase reporter construct, and firefly luciferase activity was normalized to Renilla luciferase activity in cells that had been co‐transfected with the *Nr4a1* reporter construct and the control vector. The primers for gene synthesis are listed in Table S4.

### 4.12 Chromatin immunoprecipitation (ChIP)

HEK‐293T cells were crosslinked with 1% formaldehyde (final concentration) for 10 min by inverting the flasks at room temperature and quenched with 0.125 M glycine for 5 min. The cell pellets were washed repeatedly in PBS and then stored at −80°C. The pellets were lysed in a lysis buffer (50 mM HEPES, 150 mM NaCl, 1 mM EDTA, 0.1% SDS, 0.1% sodium deoxycholate, 1% Triton X‐100, and complemented with a protease inhibitor cocktail) for 10 min. After centrifugation, the supernatant was discarded, and the pellet was lysed in a lysis buffer and subjected to sonication. The sheared chromatin was incubated with Nur77 primary antibodies or IgG bound to Pierce™ Protein A/G Agarose Beads (Thermo Fisher) overnight, followed by elution and reverse cross‐linking at 65°C overnight. A TE buffer (10 mM Tris–HCl, 1 mM EDTA) was added to DNA elution buffer followed by RNase treatment (0.5 mg/mL) at 37°C for 30 min and proteinase K treatment (0.3 mg/mL) at 51°C for 1  h, and the DNA was subsequently isolated and purified. The RT–PCR primers are listed in Table S5.

### 4.13 Statistical analysis

Data (*n* > 6) are expressed as mean ± standard error (SE), and additional data (*n* ≤ 6) are expressed as mean ± standard deviation (SD). An unpaired two‐tailed Student's *t* test and the Mann–Whitney test were used for comparisons between two groups. One‐way ANOVA coupled with the Tukey's multiple comparison test or two‐way ANOVA coupled with Sidak's multiple comparisons tests were used for comparisons of more than two groups. A value of *p* < 0.05 was considered significant. The number of replicates for each experiment is indicated in the figure legends.

## AUTHOR CONTRIBUTIONS

L.C. and D.W. designed the experiments and revised the manuscript. Y.Y. performed most of the experiments and wrote the manuscript. X.S. performed the mouse experiments and helped with manuscript writing. L.Z. G.M., W.L., and H.S. helped with primary MEF extraction and cell culture. X.W. and T.L. helped with flow cytometry. X.L. was responsible for the bioinformatic analysis. All authors discussed the results and reviewed the manuscript.

## FUNDING INFORMATION

This study was supported by Major Research Plan of the National Natural Science Foundation of China (92249305), National Key R&D Program of China (2016YFC1302400), Ministry of Education Innovation Team Development plan (IRT_17R107), National Science Foundation of China (82000827), Major Scientific and Technological Projects of Liaoning Province (2022JH1/10400001), and Talent Project for Revitalizing Liaoning, People7's Republic of China (XLYC1902013).

## CONFLICT OF INTEREST STATEMENT

The authors have declared that no conflicts of interest exist.

## Supporting information


Data S1

## Data Availability

The RNA‐sequencing data were referenced in the Gene Expression Omnibus under accession numbers GSE54714, GSE6591, GSE8150, and GSE25905. All other data supporting the findings of this study are available from the corresponding author upon reasonable request.
