# Supplementary material for: Oxidative stress impairs the Nur77‐Sirt1 axis resulting in a decline in organism homeostasis during aging
Source: Aging Cell. 2023 Mar 7;22(5):e13812. doi: 10.1111/acel.13812 (PMC10186606; doi:10.1111/acel.13812)
Supplement: Supplementary file 1 — Data S1 [file ACEL-22-e13812-s001.pdf]

Figure S1

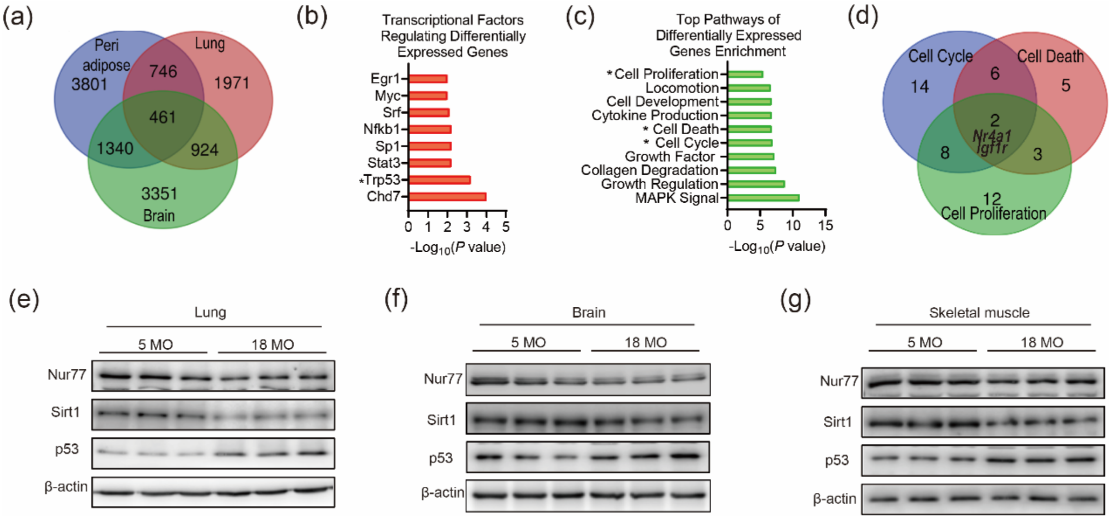

Figure S1 Nur77 is involved in regulating the aging process in multiple organs. (a) Venn diagram of differentially expressed genes in the peri-adipose, lung and brain tissues of three naturally aging mouse models. (b) The top transcription factors associated with the regulation of differentially expressed genes in multiple aging organisms as identified by TRRUST. (c) The top pathways for the regulation of differentially expressed genes in multiple aging organisms as identified by Metascape. (d) Venn diagram of differentially expressed genes associated with the cell cycle, cell death and cell proliferation in multiple aging organisms. (e) Expression of Nur77, Sirt1 and p53 in the lungs of aged mice. (f) Expression of Nur77, Sirt1 and p53 in the brains of aged mice. (g) Expression of Nur77, Sirt1 and p53 in the skeletal muscle of aged mice.

Figure S2

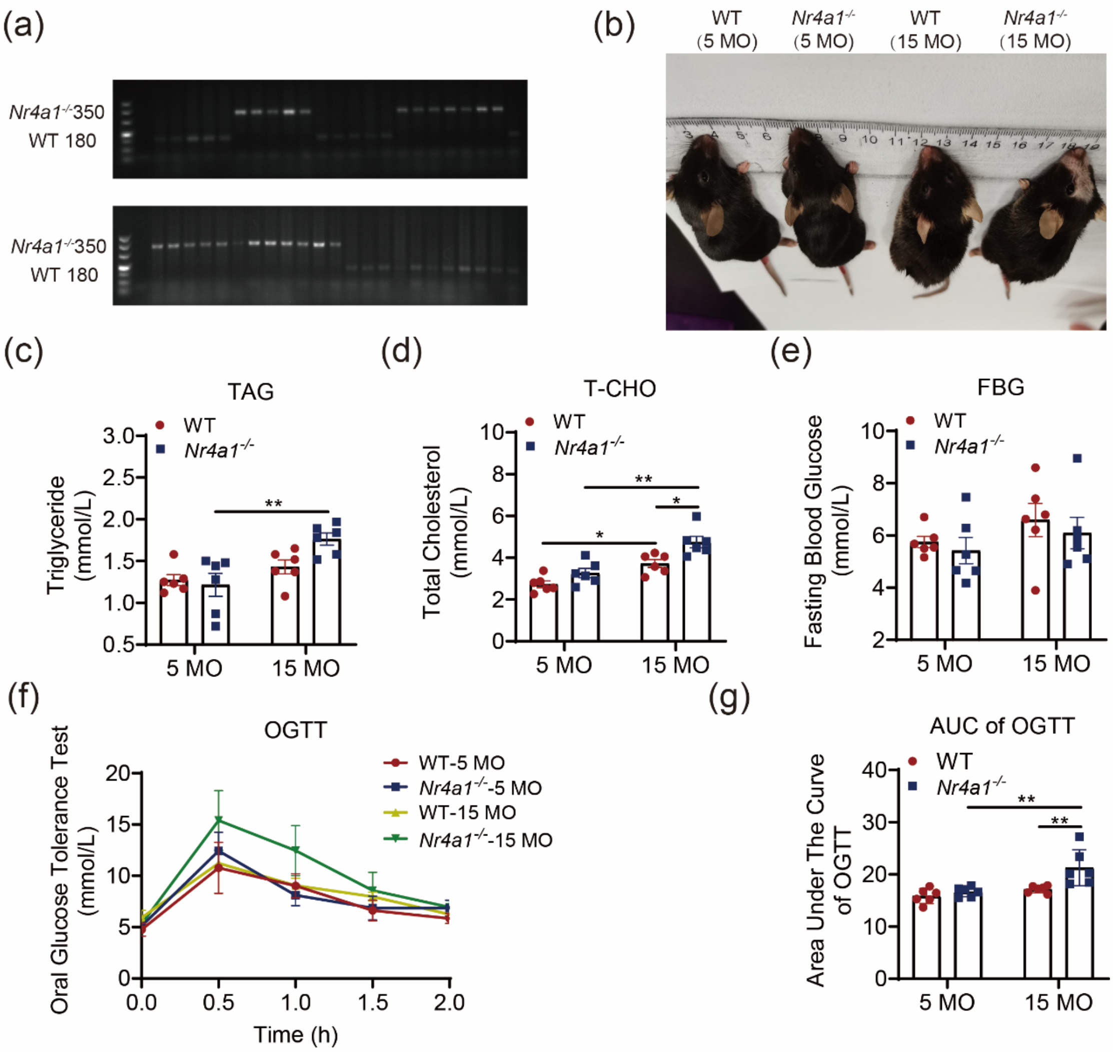

Figure S2 Nur77 deficiency results in elevated blood lipids and impaired glucose tolerance in 15-month-old mice. (a) Southern (DNA) blot of Barn HI-digested tail DNA from a litter derived by heterozygous intercross mating. The primer sequences and identification methods for mouse phenotyping were provided by Jackson Laboratory. (b) Morphology of 5- and 15-month-old WT and *Nr4a1*$^{-/-}$ mice. (c) Triglycerides (TAG) in the serum of 5- and 15-month-old WT and *Nr4a1*$^{-/-}$ mice. (d) Total cholesterol (T-CHO) in the serum of 5- and 15-month-old WT and *Nr4a1*$^{-/-}$ mice. (e) Fasting blood glucose (FBG) of 5- and 15-month-old WT and *Nr4a1*$^{-/-}$ mice. (f-g) The area under the curve (AUC) for the oral glucose tolerance test (OGTT) of 5- and 15-month-old WT and *Nr4a1*$^{-/-}$ mice.

Figure S3

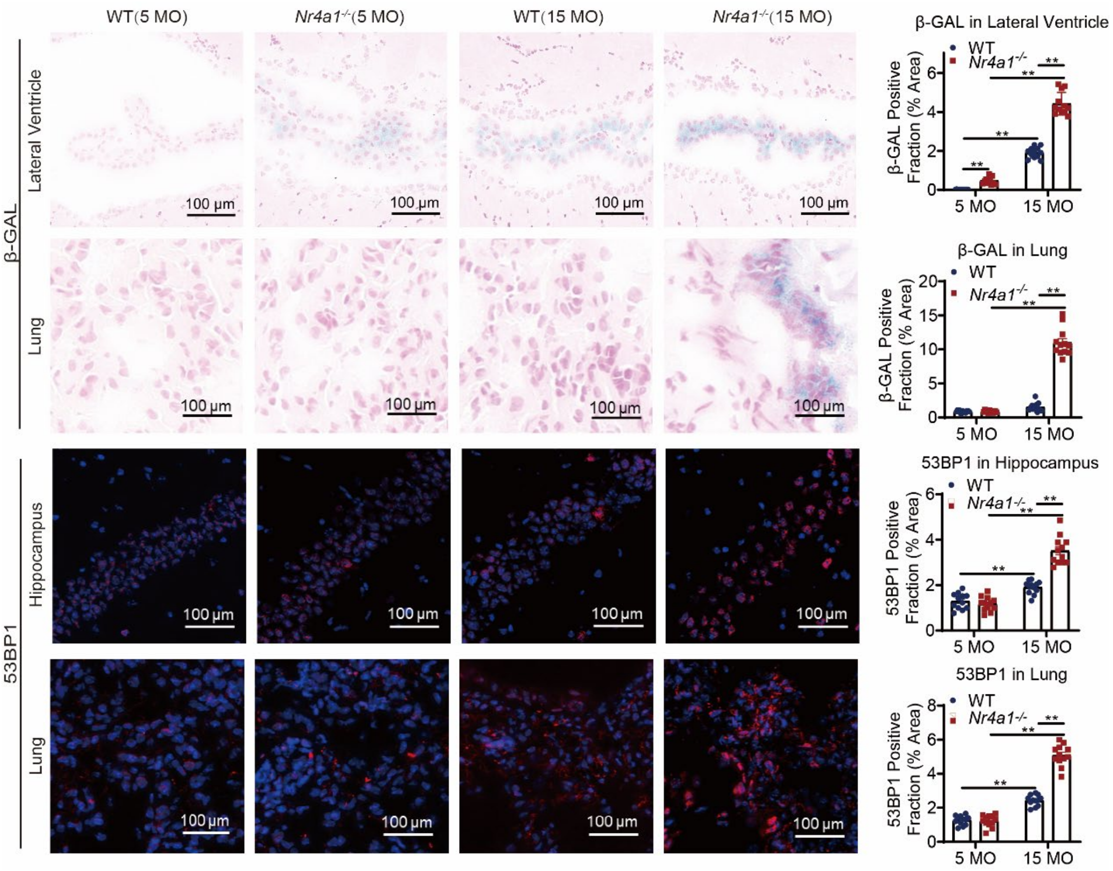

Figure S3 Nur77 deficiency increases the expression of β-galactosidase and 53BP1 in multiple tissues of 15-month-old mice. 53BP1 staining and β-galactosidase staining in the liver, kidney, brain, lung and peri-adipose tissue of 5- and 15-month-old WT and *Nr4a1*$^{-/-}$ mice. Scale bar: 100 μm. The data were analyzed by two-way ANOVA followed by multiple comparisons test. The results are plotted as the mean ± standard error. $^{**}p \leq 0.01$. 53BP1: p53 binding protein 1; β-GAL: β-galactosidase; MO: month; WT: wild-type.

Figure S4

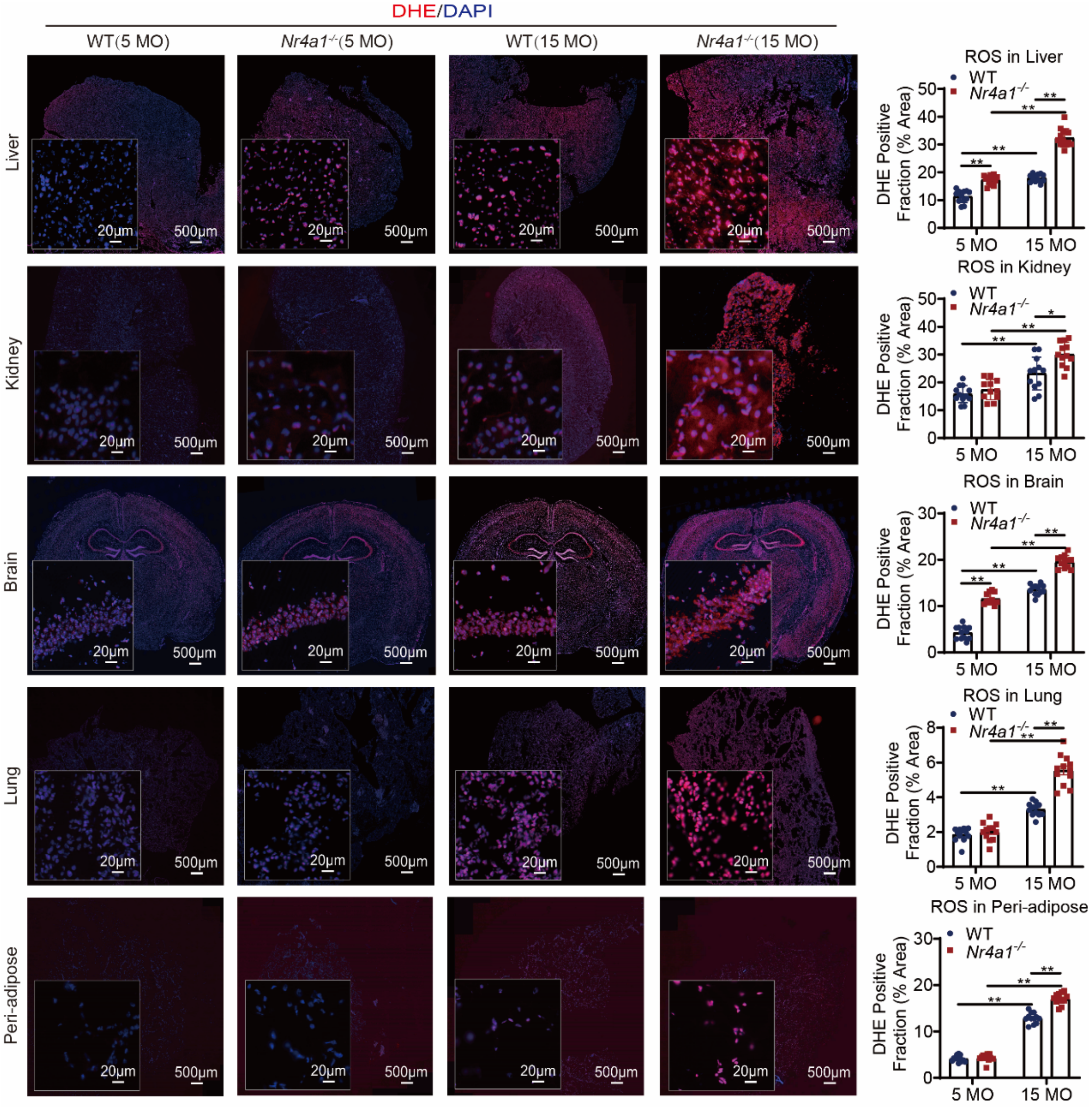

Figure S4 Nur77 deficiency increases ROS in multiple tissues of 15-month-old mice. Dihydroethidium (DHE) staining in the liver, kidney, brain, lung and peri-adipose tissue of 5- and 15-month-old WT and *Nr4a1*$^{-/-}$ mice. Scale bars are indicated for the figure and insets. The data were analyzed by two-way ANOVA followed by multiple comparisons test. The results are plotted as the mean ± standard error. *$p \leq 0.05$, **$p \leq 0.01$. DHE: dihydroethidium.

Figure S5

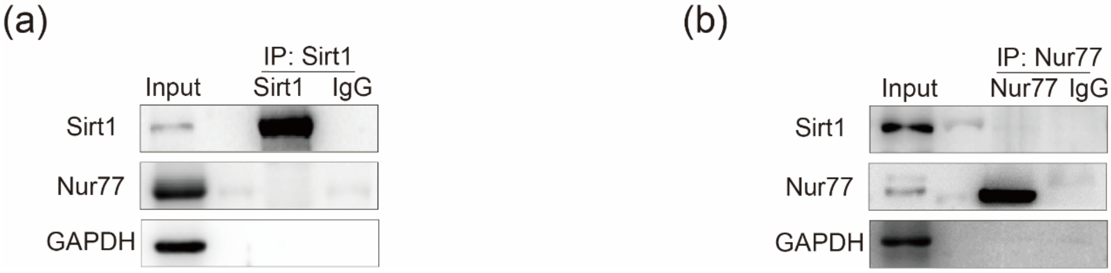

Figure S5 Nur77 does not directly interact with Sirt1. Co-IP analysis of the interaction between Nur77 and Sirt1. *n*=2 independent experiments.

Figure S6

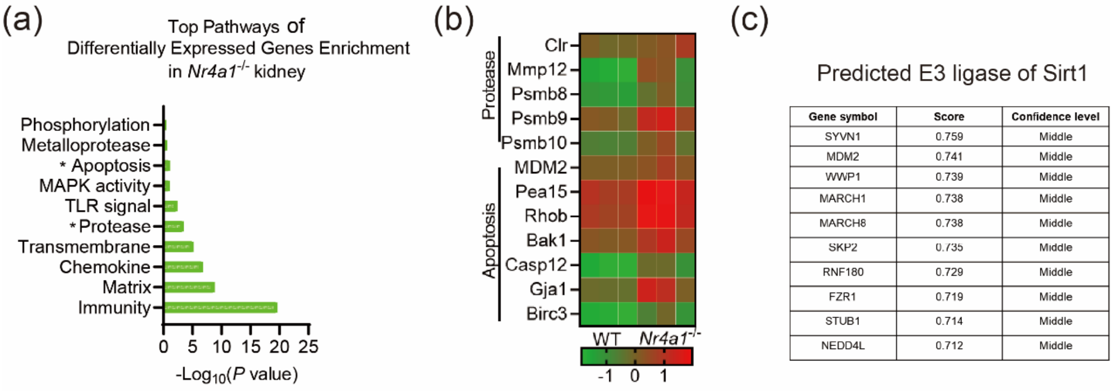

Gene symbol	Score	Confidence level
SYVN1	0.759	Middle
MDM2	0.741	Middle
WWP1	0.739	Middle
MARCH1	0.738	Middle
MARCH8	0.738	Middle
SKP2	0.735	Middle
RNF180	0.729	Middle
FZR1	0.719	Middle
STUB1	0.714	Middle
NEDD4L	0.712	Middle

Figure S6 Mechanistic analysis of Sirt1 degradation induced by Nur77 deficiency. (a) The top pathways predicted by Metascape of differentially expressed genes in the kidneys of *Nr4a1*-deficient fawn hooded hypertensive rats. (b) Heatmap of differentially expressed genes associated with proteases and apoptosis in the kidneys of *Nr4a1*-deficient fawn hooded hypertensive rats. (c) The E3 ligase prediction for Sirt1 by the UbiBrowser database.

Figure S7

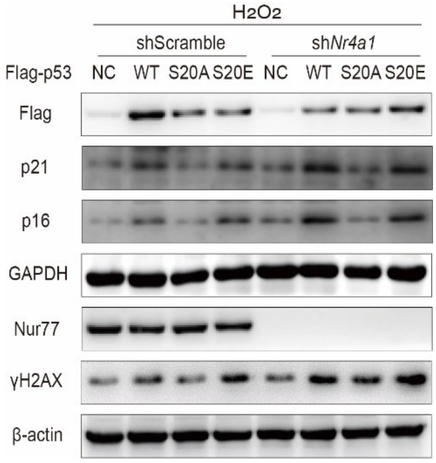

Figure S7 Nur77 regulates the expression of downstream aging-related proteins (p21, p16) through the phosphorylation of p53 at Ser20. The expression of p21, p16 and γh2AX was rescued with WT, S20A or S20E *p53* plasmids in shScramble and sh*Nr4a1* 1299 cells (p53 null). *n*=3 independent experiments.

Figure S8

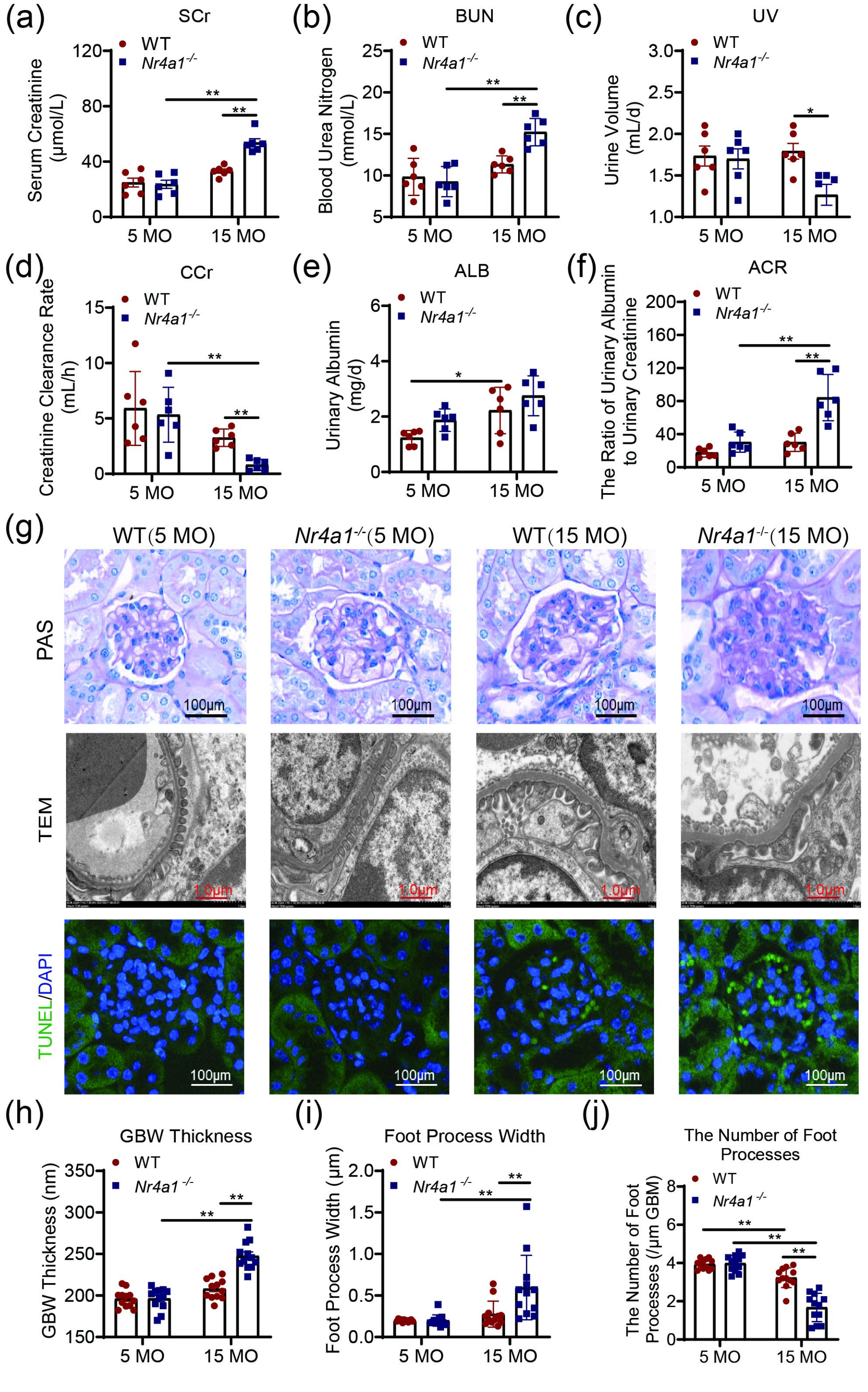

Figure S8 Nur77 deficiency aggravates functional and morphological injury in the kidney during the aging process. (a) Comparison of kidney function-related parameters associated with serum creatinine (SCr) between 5- and 15-month-old WT and *Nr4a1*$^{-/-}$

mice. (b) Comparison of kidney function-related parameters associated with blood urea nitrogen (BUN) between 5- and 15-month-old WT and *Nr4a1*$^{-/-}$ mice. (c) Comparison of kidney function-related parameters associated with urine volume (UV) between 5- and 15-month-old WT and *Nr4a1*$^{-/-}$ mice. (d) Comparison of kidney function-related parameters associated with the creatinine clearance rate (CCr) between 5- and 15-month-old WT and *Nr4a1*$^{-/-}$ mice. CCr = UV×UCr/(SCr×24×BW). (e) Comparison of kidney function-related parameters associated with urinary albumin (ALB) between 5- and 15-month-old WT and *Nr4a1*$^{-/-}$ mice. (f) Comparison of kidney function-related parameters associated with the ratio of ALB to urinary creatinine (ACR) between 5- and 15-month-old WT and *Nr4a1*$^{-/-}$ mice. (g) Morphological examination of glomeruli by periodic acid–Schiff (PAS), transmission electron microscopy (TEM) and TdT-mediated dUTP nick end labeling (TUNEL) staining in the kidneys of 5- and 15-month-old WT and *Nr4a1*$^{-/-}$ mice. Scale bars are indicated. (h) Analyses of glomerular basement membrane (GBM) thickness in the kidneys of 5- and 15-month-old WT and *Nr4a1*$^{-/-}$ mice. (i) Analyses of foot process width in the kidneys of 5- and 15-month-old WT and *Nr4a1*$^{-/-}$ mice. (j) Analyses of the number of foot processes per μm of GBM in the kidneys of 5- and 15-month-old WT and *Nr4a1*$^{-/-}$ mice. Six glomeruli per mouse were analyzed, $n = 2$ mice per group. The data were analyzed by two-way ANOVA followed by multiple comparisons test. The results are plotted as the mean ± standard error. $^{**}p \leq 0.01$.

Figure S9

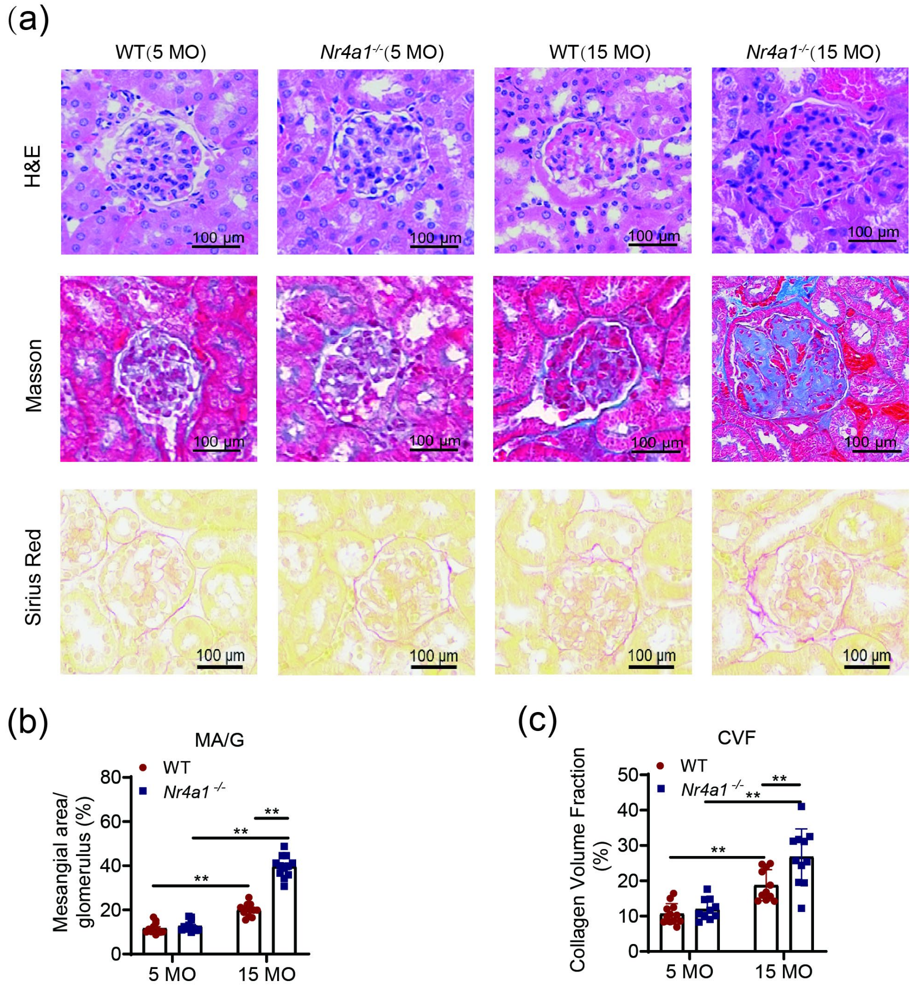

Figure S9 Nur77 deficiency aggravates morphological injury in the kidney during the aging process. (a) Comparison of kidney structure between wild-type (WT) and *Nr4a1*$^{-/-}$ mice in the 5 MO and 15 MO mouse groups by hematoxylin–eosin (H&E), Masson and Sirius red staining. (b) Analyses of mesangial area glomeruli (MA/G) in the kidneys of 5- and 15-month-old WT and *Nr4a1*$^{-/-}$ mice. (c) Analyses of the collagen volume fraction (CVF) in the glomerulus in 5- and 15-month-old WT and *Nr4a1*$^{-/-}$ mice. The data were analyzed by two-way ANOVA followed by multiple comparisons test. The results are plotted as the mean ± standard error. $^{**}p \leq 0.01$. CVF: collagen volume fraction, H&E: hematoxylin–eosin, MA/G: mesangial area glomerulus, MO: month-old.

Figure S10

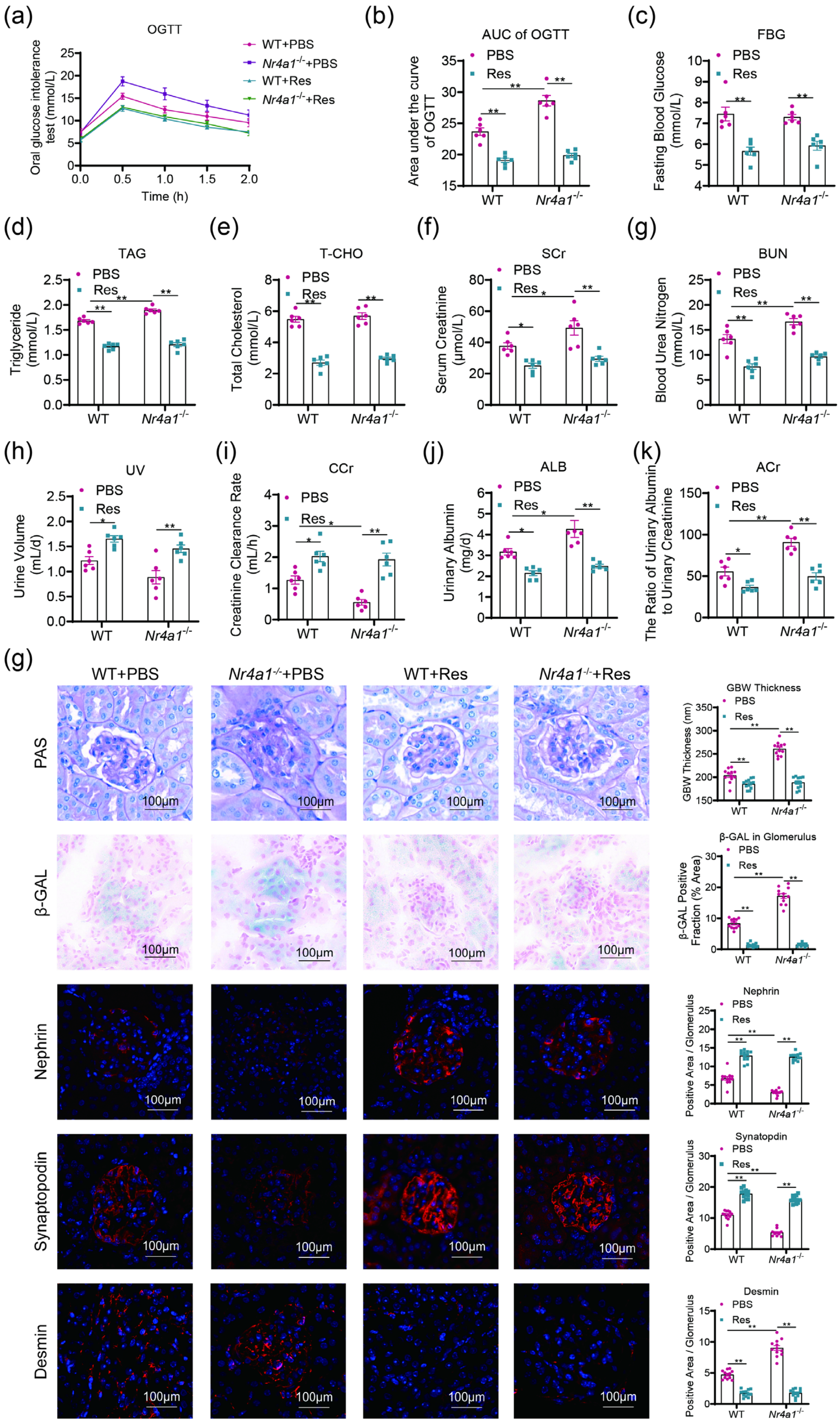
(a)
OGTT
WT+PBS
Nr4a1-/-+PBS
WT+Res
Nr4a1-/-+Res
Oral glucose intolerance test (mmol/L)
Time (h)
(b)
AUC of OGTT
Area under the curve of OGTT
(c)
FBG
Fasting Blood Glucose (mmol/L)
(d)
TAG
Triglyceride (mmol/L)
(e)
T-CHO
Total Cholesterol (mmol/L)
(f)
SCr
Serum Creatinine (μmol/L)
(g)
BUN
Blood Urea Nitrogen (mmol/L)
(h)
UV
Urine Volume (mL/d)
(i)
CCr
Creatinine Clearance Rate (mL/h)
(j)
ALB
Urinary Albumin (mg/d)
(k)
ACr
The Ratio of Urinary Albumin to Urinary Creatinine
PBS
Res
WT
Nr4a1-/-
(g)
WT+PBS
Nr4a1-/-+PBS
WT+Res
Nr4a1-/-+Res
PAS
β-GAL
Nephrin
Synaptopodin
Desmin
100μm
GBW Thickness
GBW Thickness (nm)
β-GAL in Glomerulus
β-GAL Positive Fraction (% Area)
Nephrin
Synatopdin
Desmin
Positive Area / Glomerulus

Figure S10 Resveratrol ameliorates kidney damage in aged WT and *Nr4a1*$^{-/-}$ mice. (a-b) The area under the curve (AUC) for the oral glucose tolerance test (OGTT) of aged WT and *Nr4a1*$^{-/-}$ mice with or without resveratrol treatment. (c) Fasting blood glucose (FBG) of aged WT and *Nr4a1*$^{-/-}$ mice with or without resveratrol treatment. (d) Triglycerides (TAG) in the serum of aged WT and *Nr4a1*$^{-/-}$ mice with or without resveratrol treatment. (e) Total cholesterol (T-CHO) in the serum of aged WT and *Nr4a1*$^{-/-}$ mice with or without resveratrol treatment. (f) Comparison of kidney function-related parameters associated with serum creatinine (SCr) between resveratrol treated and untreated aged WT and *Nr4a1*$^{-/-}$ mice. (g) Comparison of kidney function-related parameters associated with blood urea nitrogen (BUN) between resveratrol treated and untreated aged WT and *Nr4a1*$^{-/-}$ mice. (h) Comparison of kidney function-related parameters associated with urine volume (UV) between resveratrol treated and untreated aged WT and *Nr4a1*$^{-/-}$ mice. (i) Comparison of kidney function-related parameters associated with the creatinine clearance rate (CCr) between resveratrol treated and untreated aged WT and *Nr4a1*$^{-/-}$ mice. (k) Comparison of kidney function-related parameters associated with urinary albumin (ALB) between resveratrol treated and untreated aged WT and *Nr4a1*$^{-/-}$ mice. (g) Morphological examination of glomeruli by periodic acid–Schiff (PAS), β-galactosidase staining (β-GAL) and immunofluorescence staining of nephrin, synaptopodin and desmin. Six glomeruli per mouse were analyzed, $n = 2$ mice per group. The data were analyzed by two-way ANOVA followed by multiple comparisons test. *$p \leq 0.05$, **$p \leq 0.01$.

Table S1. The predicted sequence of Nur77 that binds to *MDM2*

**Name**	**Score**	**Relative score**	**Sequence ID**	**Start**	**End**	**Strand**	**Predicted sequence**
Nur77	11.1	0.926284905	NC_000012.12:68806172-68808172	891	900	+	AAAAGGTCCT
Nur77	10.9222	0.907163953	NC_000012.12:68806172-68808172	219	230	-	TAAAAGTTCAGA
Nur77	7.21789	0.856049805	NC_000012.12:68806172-68808172	220	229	-	AAAAGTTCAG
Nur77	7.99403	0.852498727	NC_000012.12:68806172-68808172	890	901	+	AAAAAGGTCCTT
Nur77	7.91603	0.851042584	NC_000012.12:68806172-68808172	252	263	-	TTAAATATCAAG
Nur77	6.01953	0.834369246	NC_000012.12:68806172-68808172	1357	1366	-	TTGAGGTCAG
Nur77	7.00062	0.83395311	NC_000012.12:68806172-68808172	713	724	+	TTAAAAGCCACA
Nur77	6.88637	0.831820381	NC_000012.12:68806172-68808172	627	638	-	GAAAATCTCAAG
Nur77	6.8535	0.831206641	NC_000012.12:68806172-68808172	263	274	+	ACAAATATCAAC
Nur77	5.26813	0.820775026	NC_000012.12:68806172-68808172	10	19	-	TGAAAGTCAA
Nur77	6.23162	0.819596975	NC_000012.12:68806172-68808172	1446	1457	+	TAAAAGCGCAGA
Nur77	6.11598	0.817438252	NC_000012.12:68806172-68808172	9	20	-	ATGAAAGTCAAG
Nur77	5.61213	0.808031996	NC_000012.12:68806172-68808172	1555	1566	-	AAAAAGATTAAA

Nur77	5.59567	0.807724677	NC_000012.12:68806172-68808172	609	620	-	GAAAATGTAAGC
Nur77	5.48087	0.805581648	NC_000012.12:68806172-68808172	169	180	-	ACAAAGGCCTCA
Nur77	4.18274	0.801138389	NC_000012.12:68806172-68808172	170	179	-	CAAAGGCCTC

Table S2. Antibodies used in the co-immunoprecipitation and western blot analyses

Antibody	Cat. No.	Company
Anti-Nur77	NB100-56745	NOVUS
Anti-Sirt1	S5447	Sigma–Aldrich
Anti-MDM2	sc-965	Santa Cruz
Anti-Flag	ab1162	Abcam
Anti-GFP	2955S	Cell Signaling
Anti-HA	3724S	Cell Signaling
Anti-Ubiquitin	3933	Cell Signaling
Anti-p53 (acetyl K370)	ab183544	Abcam
Anti-p53 (acetyl K382)	ab75754	Abcam
Anti-p53 (phospho S15)	ab1431	Abcam
Anti-p53 (phospho S20)	9287S	Cell Signaling
Anti-p21	ab109199	Abcam
Anti-p16	ab51243	Abcam
Anti-Bax	ab32503	Abcam
Anti-Bcl2	ab182858	Abcam
Anti-γh2AX (phospho S139)	9718	Cell Signaling
Anti-53BP1	NB100-304	NOVUS
Anti-Lysine (Ac-K)	ab80178	Abcam
Anti-ATM	2873	Cell Signaling
Anti-ATM (phospho S1981)	13050	Cell Signaling
Anti-Chk2	6334	Cell Signaling
Anti-Chk2 (phospho T68)	2197	Cell Signaling
Anti-Nephrin	ab216341	Abcam
Anti-Synaptopodin	21064-1-AP	Proteintech
Anti-Desmin	16520-1-AP	Proteintech
Anti-GAPDH	60004-1-Ig	Proteintech
Anti-β-Actin	66009-1-Ig	Proteintech

Table S3. Primers used for quantitative real-time polymerase chain reaction (PCR) assays

Genes	Forward primer	Reverse primer
mmu-*Sirt1*	CGCTGTGGCAGATTGTTATTAA	TTGATCTGAAGTCAGGAATCCC
mmu-*MDM2*	CCAGGCCAATGTGCAATACC	ATGGTTTTGGTCTAACCAGAGT
mmu-*GAPDH*	ACCCTTAAGAGGGATGCTGC	CCCAATACGGCCAAATCCGT
hsa-*Sirt1*	TAGCCTTGTCAGATAAGGAAGGA	ACAGCTTCACAGTCAACTTTGT
hsa-*MDM2*	GAATCATCGGACTCAGGTACATC	TCTGTCTCACTAATTGCTCTCCT
has-*GAPDH*	TCGGAGTCAACGGATTTGGT	TTCCCGTTCTCAGCCTTGAC

Table S4. Primers for gene synthesis used in double luciferase experiments

Genes	Forward primer	Reverse primer
WT1-<br>*MDM2*	AGAACATTTCTCTATCGATAGG<br>TACCGGAAGTTTCCTTTCTGG<br>TAGGCTGGGAAGCGGG	AGCTTACTTAGATCGCAGAT<br>CTCGAGGAATTGAGTTAGCT<br>AAACCCAAAAACACAGC
MUT1-<br>*MDM2*	AGAACATTTCTCTATCGATAGG<br>TACCGGAAGTTTCCTTTCTGG<br>TAGGCTGGGAAGCGGG	AGCTTACTTAGATCGCAGAT<br>CTCGAGGAATTGAGTTAGCT<br>AAACCCAAAAACACAGC
WT2-<br>*MDM2*	AGAACATTTCTCTATCGATAGG<br>TACCCTTTTATTTAAGTCTCAC<br>CATCTTTCAAGACAC	AGCTTACTTAGATCGCAGAT<br>CTCGAGTCCATCTCAGAAGA<br>AAAAAAAAAAAAAAGAGA
MUT2-<br>*MDM2*	AGAACATTTCTCTATCGATAGG<br>TACCCTTTTATTTAAGTCTCAC<br>CATCTTTCAAGACAC	AGCTTACTTAGATCGCAGAT<br>CTCGAGTCCATCTCAGAAGA<br>AAAAAAAAAAAAAAGAGA
Nr4a1	TTAAGCTTGGTACCGAGCTCG<br>GATCCATGACAAGTGCACAGT<br>ATAAAATCAAGATTCTA	GTTTAAACGGGCCCTCTAGA<br>CTCGAGTCAGAAGGGCAGC<br>GTGTCCATGAAGATCTTGTC

Table S5. Primers used for quantitative real-time polymerase chain reaction (PCR) in chromatin immunoprecipitation (ChIP) assays

Genes	Forward primer	Reverse primer
MDM2 S1-1	GCTGCAGAAGGGAAGGATATAA	CACAGCCATTGCAAAGAAGG
MDM2 S1-2	GCGGGAGAAGGGAAGGATATAA	CTCTGGCCAGTAAGTGATTAGC
MDM2 S1-3	AAAGCTGCAGAAGGGAAG	GGAACACGTTTCTTCTCTGG
MDM2 S2-1	GAACTGCCTCCAGCATTTACTA	CAAGTGAACGAATGGGTTTGTG
MDM2 S2-2	CCTCCAGCATTTACTAAGCAATTA	TGGGTGACAGAGCAAGA
MDM2 S2-3	GAACTGCCTCCAGCATTTACTA	CAAGTGAACGAATGGGTTTGTG
